# Characterizing the sensorimotor domain in schizophrenia spectrum disorders

**DOI:** 10.1007/s00406-021-01354-9

**Published:** 2021-11-27

**Authors:** Stefan Fritze, Fabio Sambataro, Katharina M. Kubera, Geva A. Brandt, Andreas Meyer-Lindenberg, Robert C. Wolf, Dusan Hirjak

**Affiliations:** 1 Department of Psychiatry and Psychotherapy, Central Institute of Mental Health, Medical Faculty Mannheim, Heidelberg University, Mannheim, Germany; 2 Department of Neuroscience (DNS), University of Padova, Padova, Italy; 3 Padova Neuroscience Center, University of Padova, Padua, Italy; 4 Center for Psychosocial Medicine, Department of General Psychiatry, University of Heidelberg, Heidelberg, Germany

**Keywords:** Sensorimotor dysfunction, Schizophrenia spectrum disorders, NSS, Dyskinesia, Parkinsonism, Akathisia, Catatonia

## Abstract

The rapidly evolving field of sensorimotor neuroscience reflects the scientific and clinical relevance of sensorimotor abnormalities as an intrinsic component of the disease process, e.g., in patients with schizophrenia spectrum disorders (SSD). Despite previous efforts, however, prevalence rates and relationships between different categories of sensorimotor abnormalities in SSD patients are still subject of ongoing debate. In this study, we examined five different categories of the sensorimotor domain (Neurological soft signs (NSS), parkinsonism, catatonia, akathisia, and tardive dyskinesia) according to well-established clinical ratings scales and the respective cut-off criteria in a sample of 131 SSD patients. We used a collection of statistical methods to better understand prevalence, overlap and heterogeneity, as well as psychopathological and cognitive correlates of sensorimotor abnormalities. 97.7% of the SSD patients considered by this study exhibited at least one categorically defined sensorimotor abnormality that tended to co-vary within three different sensorimotor subgroups (moderate, hyperkinetic and hypokinetic). Finally, hyperkinetic and hypokinetic groups differed significantly in their neurocognitive performance compared with the moderate group. The results suggest different patterns of clinical overlap, highlight the relationship between sensorimotor and cognitive domain and provide clues for further neurobiological studies.

**Supplementary Information:**

The online version contains supplementary material available at 10.1007/s00406-021-01354-9.

## Introduction

The growing interest in movement disorder as well as sensorimotor and psychomotor functioning in schizophrenia spectrum disorders (SSD) and other psychiatric disorders [1–4] has been reflected by the recent introduction of the “sensorimotor domain” in the research domain criteria (RDoC) matrix, as developed and promoted by the National Institute of Mental Health (NIMH) [5]. A number of studies have demonstrated the presence of Neurological Soft Signs (NSS), catatonia, parkinsonism, akathisia and tardive dyskinesia (TD) in SSD patients [2, 6, 7]. These sensorimotor abnormalities have been convincingly shown to be linked to the disease process by their presence in both antipsychotic-naïve SSD patients [6, 7] and their first-degree relatives [8–11]. Furthermore, magnetic resonance imaging (MRI) studies have demonstrated that sensorimotor abnormalities are associated with structural and functional changes within the cortical–thalamic–cerebellar–cortical circuit (CTCC), which is intricately linked to SSD itself [12, 13]. In line with this, sensorimotor abnormalities appear to be intrinsic to SSD in a manner that can be improved or exacerbated [7, 14] by antipsychotic medication [4, 15].

From a clinical perspective, sensorimotor abnormalities can be characterized by specific symptom patterns, that is, specific constellations of NSS, catatonia, parkinsonism, akathisia and TD. This means that patients can show distinct and overlapping symptoms/patterns of different sensorimotor categories. Further characterization of sensorimotor abnormalities in SSD and their covariation may improve future diagnosis and treatment efforts, particularly when taking into account that distinct sensorimotor abnormalities have also been proposed as possible predictors of treatment response [14, 16].

In the present study, we sought to examine prevalence, overlap and heterogeneity, as well as psychopathological and cognitive correlates of a broad spectrum of sensorimotor abnormalities in a well-characterized sample of 131 SSD patients. For this purpose, we combined different statistical methods based on clinical and psychopathological data to deeply characterize sensorimotor abnormalities and their psychopathological correlates in SSD. In the first step, we sought to assess the prevalence of NSS, catatonia, parkinsonism, akathisia and TD in SSD patients. Based on previous literature on antipsychotic-naïve and treated SSD patients we predicted the following pattern (continuum) of prevalence rates in our sample: NSS > parkinsonism > catatonia > TD > akathisia [17]. In the second step, we were interested in defining the categories with the greatest symptom overlap and comparing them with previous literature. Based on recent data, including own studies [16, 18–20], we hypothesized that there will be an overlap between NSS and parkinsonism and catatonia and parkinsonism. Since the differentiation between parkinsonism, catatonia, psychomotor slowing and negative symptoms is an important topic in SSD research, in a third step we sought to clarify the relationship between parkinsonism, catatonia, psychomotor slowing and negative symptoms in our sample. Finally, we hypothesized that there would be evidence of different sensorimotor subgroups in SSD based on sensorimotor abnormalities [42]. We were specifically interested in addressing this empirically to determine: (1) whether specific subgroups exist; (2) the optimal number of subgroups that explain the heterogeneity; and (3) the clinical, functional and cognitive correlates associated with each sensorimotor subgroup.

## Methods

### Study participants

We evaluated a total of 131 right- and left-handed [21] patients according to DSM-IV-TR [22] criteria for schizophrenia (*n* = 119), schizoaffective disorder (*n* = 7) and schizotypal personality disorder (*n* = 5) [23, 24]. Diagnoses were made by staff psychiatrists and confirmed using the German versions of the Structured Clinical Interview for DSM-IV-TR axis I and II disorders (SCID) and examination of the case notes (D.H. and S.F.) (see supplementary material for exclusion criteria). The local Ethics Committee (Medical Faculty at Heidelberg University, Germany) approved the study. We obtained written informed consent from all study participants after all aims and procedures of the study had been fully explained.

### Clinical assessment

All study participants were examined during inpatient treatment as soon as possible after partial remission of acute psychopathological symptoms. All relevant study procedures (e.g. psychometric testing, motor assessment) were completed within 3 days. None of the SSD patients were treated with benzodiazepines or anticholinergic agents at the time of the psychometric testing and sensorimotor assessment. Patients received antipsychotic medication according to their psychiatrist’s choice. All but 9 patients (9/131 = 6.8%) were on a stable daily dose of antipsychotic medication for at least 14 days. 81 patients (81/131 = 61.8%) were receiving monotherapy (aripiprazole: *n* = 17; olanzapine: *n* = 15; quetiapine: *n* = 12; amisulpride: *n* = 11; clozapine: *n* = 9; risperidone: *n* = 8; paliperidone: *n* = 7; flupenthixol: *n* = 1; haloperidol: *n* = 1) and 41 patients (41/131 = 31.2%) were receiving a combination of two antipsychotics. Twenty-six patients (26/131 = 19.84%) received clozapine (9 were on monotherapy and 17 were receiving clozapine combined with a second-generation antipsychotic). Only four patients (4/131 = 3.0%) were receiving first-generation antipsychotics. The daily doses of antipsychotic medication were converted to olanzapine equivalents (OLZ) [25]. The evaluation of psychopathology was performed with Positive and Negative Syndrome Scale [PANSS] (test–retest reliability across a 3- to 6-month inpatient phase [*r* = 0.80,0.68, and 0.60] [26]) [27], Brief Psychiatric Rating Scale [BPRS] (reliability: *r* = 0.78, *p* < 0.001 [28]) [29], Clinical Global Impression Scale [CGI] (admission vs. discharge CGI-S scores: *r* = 0.40 [30]) [31] and Global assessment of functioning [GAF] (inter-rater reliability: *r* = 0.26 [32]) [33]. The examination of executive functioning and processing speed was performed with Trail-Making-Test B (TMT-B) and Digit Symbol Substitution Test (DSST) as two tests of the Brief Cognitive Assessment Tool for Schizophrenia (B-CATS) (intraclass correlation coefficient: 0.82) [34]. For the examination of sensorimotor abnormalities, we employed the following rating scales: NSS: Heidelberg NSS Scale (test–retest reliability in healthy subjects [*r* = 0.80, *df* = 20, *p* < 0.001] [35]) [36]; parkinsonism: Simpson and Angus Scale (SAS) (test–retest reliability not available; inter-rater reliability: *r* = 0.71–0.96) [37]; catatonic symptoms: Northoff Catatonia Rating Scale (NCRS) [38]; akathisia: Barnes Akathisia Rating Scale (BARS) (inter-rater reliability: *κ* = 0.74–0.95) [39, 40]; and TD: Abnormal Involuntary Movement Scale (AIMS) (internal consistency = 0.05–0.29) [41]. For detailed description of the sensorimotor rating scales and the threshold values see supplementary material. Finally, the severity of psychomotor slowing was determined using the corresponding BPRS item #13 [29]. All clinical and sensorimotor rating scales were performed by two raters (SF and DH), who reached an intraclass correlation coefficient > 0.85.

### Statistical analyses

We used SPSS for Windows version 26 and RStudio 1.3.1093. Initially, a descriptive analysis for demographic and clinical data in SSD patients (Table 1) was performed.

**Table 1:** Clinical and demographic variables in schizophrenia spectrum disorders (SSD; *n* = 131) patients

Variable	
Age (years)	38.31 ± 11.65
Gender (m/f)	73/58
Education (years)	13.05 ± 2.85
Packyears	6.24 ± 23.81
Olanzapine equivalents	17.53 ± 10.52
Duration of illness (years)	10.60 ± 11.04
*PANSS total score*	67.10 ± 20.99
*PANSS positive score*	15.28 ± 6.83
*PANSS negative score*	16.93 ± 7.67
*PANSS global score*	34.96 ± 10.86
*BPRS total score*	37.34 ± 12.78
*BPRS item #13*	1.98 ± 1.24
*GAF score*	69.38 ± 17.04
*CGI-S*	3.85 ± .99
*NCRS motor score*	.68 ± 1.07
*NCRS affective score*	1.57 ± 1.77
*NCRS behavior score*	.82 ± 1.23
*NCRS total score*	2.97 ± 3.27
*SAS total score*	2.77 ± 2.56
*AIMS total score*	1.05 ± 2.39
*BARS global score*	.89 ± 1.29
*TMT-B*	113.74 ± 66.6

Data are mean ± standard deviation

*PANSS* Positive and Negative Symptoms Scale (p = positive, n = negative, g = global), *BPRS* Brief Psychiatric Rating Scale, *BPRS item #13* psychomotor slowing, *GAF* Global Assessment of Functioning, *CGI-S* Clinical Global Impression Scale (Severity), *SAS* Simpson and Angus Scale, *AIMS* Abnormal involuntary movement scale, *BARS* Barnes Akathisia Rating Scale, *NCRS* Northoff Catatonia Rating Scale, *TMT-B* Trail-Making-Test B

In a first step, we examined the prevalence rates of the five categories of sensorimotor abnormalities. In the second step, we explored the overlap between the five categories of sensorimotor abnormalities using pre-defined cut-off values to define the presence of each sensorimotor abnormality in each patient. RStudio was used to create a Venn diagram using these data. The idea of this type of diagram is that different classes can be represented in such relation to each other and that all possible logical relations of these classes can be shown in the same diagram. Venn diagrams contain overlapping areas. The interior of the area represents the elements which are part of the set, while the exterior represents elements that are not part of the set. In the third step, to examine the relationship between parkinsonism and catatonia in the whole sample (*n* = 131), we ran a partial correlation (two-tailed) between an individual’s SAS and NCRS scores while controlling for age, gender, OLZ and PANSS-N. Further, we ran a partial correlation (two-tailed) between an individual’s SAS and NCRS total scores and BPRS item #13 and PANSS-N scores while controlling for age, gender, and OLZ to determine the relationship between parkinsonism, catatonia, psychomotor slowing and negative symptoms in the whole sample (*n* = 131). *P* values of the identified associations were corrected for the number of clinical assessments in our main analysis using the Bonferroni method. For this reason, the corrected threshold was set to *p* = 0.016 [*α* = 0.05/3 tests (1 SAS total score vs. 1 NCRS total score + 1 SAS total scores vs. 1 PANSS-N score + 1 NCRS total score vs. PANSS-N score)].

In a fourth step, in order to identify homogeneous subgroups of SSD patients based on their sensorimotor dysfunction, we conducted a hierarchical cluster analysis [similar to Burdick et al. 42]. Similarity between cases was computed with the squared Euclidian distance and complete linkage was selected as the agglomeration procedure [42]. Since the variables (scores for each sensorimotor abnormality) did not have the same metrics, pre-standardization using Z-transformation was necessary. According to visual scrutiny of the resulting dendrogram, the appropriate number of clusters was selected. With the purpose of testing the validity of the clusters, silhouette method [43] was chosen (see supplementary materials). Subsequently, a linear discriminant function analysis (DFA) was conducted. The DFA explored the predictive power of the five sensorimotor categories in separating into the discrete sensorimotor groups acquired by the hierarchical cluster analysis. Also, in order to test reliability, DFA was repeated in a split-half of the sample (s. supplementary materials). Fifth, to ascertain if demographic and clinical variables (age, gender, duration of illness and OLZ) differed among sensorimotor clusters, we performed analysis of variance (ANOVA). To compare functional (GAF), cognitive (TMT-B and DSST) and psychopathological scales (PANSS) between sensorimotor clusters, we performed two-way analyses of covariance (ANCOVA) models adjusted for age, gender, education and OLZ. Finally, multiple linear regression analyses were run to determine the relationship between composite sensorimotor (CSM) scores (calculated as mean from z-standardized AIMS, BARS, NCRS, NSS and SAS scores) and demographic (age, gender, duration of illness and OLZ), functional (GAF), cognitive (TMT-B and DSST) and psychopathological variables (PANSS) in the whole patient sample. If appropriate, post-hoc pair-wise *t*-tests were conducted, with α-significance level correction for multiple testing (0.05/9 = 0.005).

Last, to exclude that the results in SSD patients were unduly driven by a total of 7 schizoaffective and 5 schizotypal disorder patients, we rerun the analyses in Sect. 3.2., 3.3., 3.4., 3.5. and 3.6. with the exclusion of these patients to create a more homogeneous sample (*n* = 119) with regard to diagnostic status.

## Results

### Clinical and demographic characteristics

Demographic and clinical characteristics of the study group comprising 131 subjects (73 male / 58 female) are shown in Table 1. Descriptive statistics of OLZ values yielded the following results: minimum: 0.0; 25% percentile: 10.40; median: 15.70; 75% percentile: 22.60; maximum: 48.20; range: 0.0–48.20; Std. error of mean: 0.91; lower 95% CI of mean 15.72; upper 95% CI of mean: 19.35; coefficient of variation: 60.4%.

### Prevalence and overlap of sensorimotor abnormalities

**Fig. 1:**
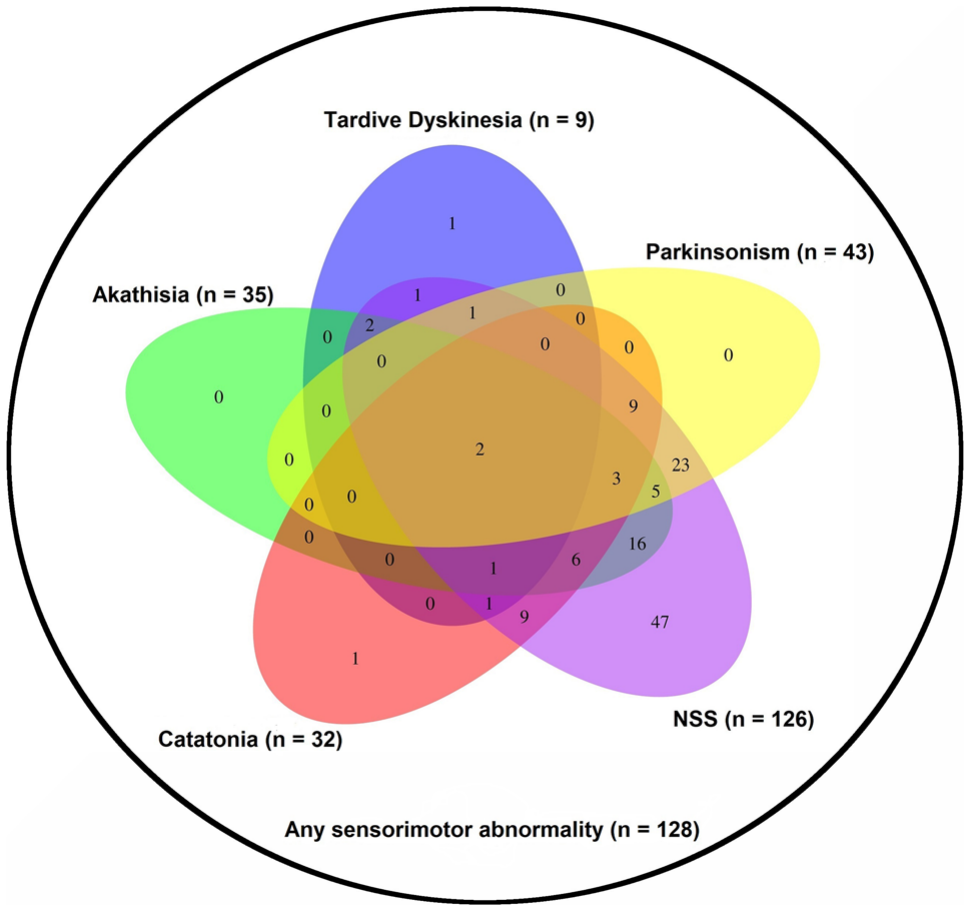
Venn diagram. Each sensorimotor category is represented by a colored oval. Overlapping regions show the number of patients exhibiting the respective sensorimotor categories (according to predefined thresholds). Numbers in non-overlapping portions of each oval show the number of patients exhibiting unique sensorimotor category (according to predefined thresholds)

The highest prevalence (126 subjects, 96%) was detected for NSS (Fig. 1). In addition, 43 (32.8%) subjects were defined to satisfy SAS criteria for parkinsonism, while 35 subjects (26.7%) had akathisia according to BARS criteria. Also, 32 subjects (24.4%) were defined as having catatonia according to NCRS (Fig. 1). Finally, nine subjects (6.9%) satisfied TD criteria of Schooler–Kane, 128 patients (97.7%) satisfied criteria for at least one sensorimotor abnormality, two patients (1.5%) fulfilled criteria of all five neuromotor scores. The largest overlap of patients fulfilling cut-off criteria for a distinct sensorimotor category were found between NSS and parkinsonism (*n* = 43, 32.8%), NSS and catatonia (*n* = 31, 23.7%), NSS and akathisia (*n* = 35, 26.7%), catatonia and parkinsonism (*n* = 14, 10.7%) and between catatonia and akathisia (*n* = 12, 9.1%) (Fig. 1 and supplementary table 1). After the exclusion of 7 schizoaffective and 5 schizotypal disorder patients, the analyses confirmed most of our findings (see supplementary material for results).

### Catatonia, parkinsonism, psychomotor slowing and negative symptoms

**Fig. 2:**
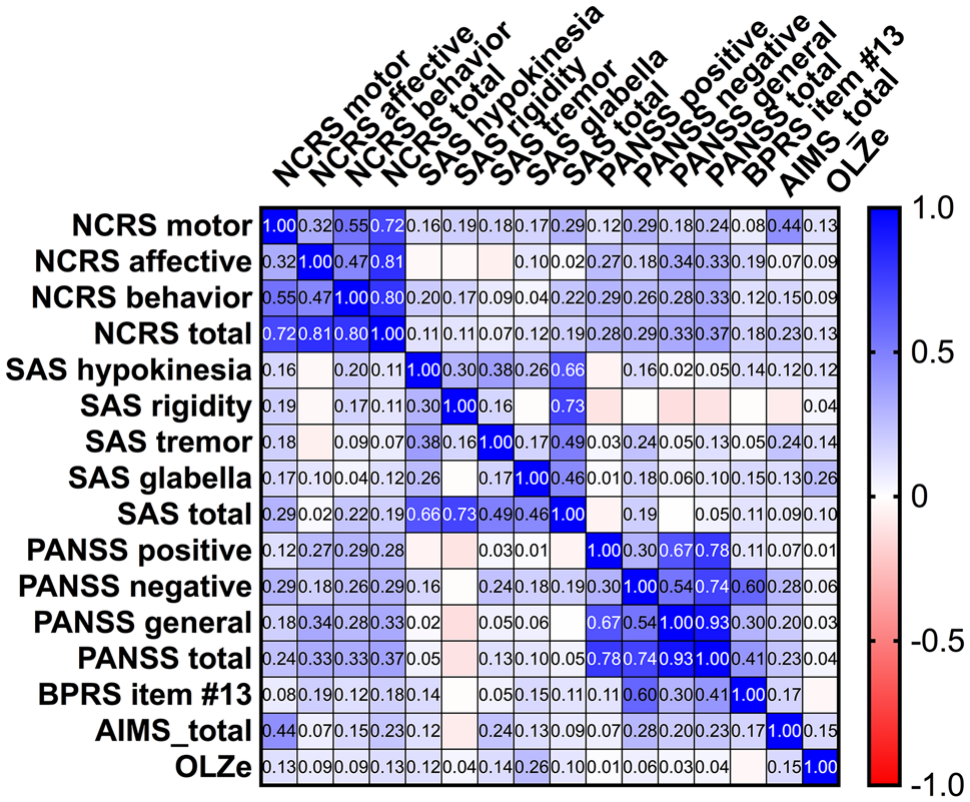
Graphical Pearson correlation matrix of sensorimotor abnormalities, psychopathological symptoms and OLZ. Pearson correlation r values were determined using GraphPad Prism 9. Colors are added for better visualization. The colors span from dark blue to dark red, where dark blue denotes a r value of 1, and dark red indicates a r value of -1

**Fig. 3:**
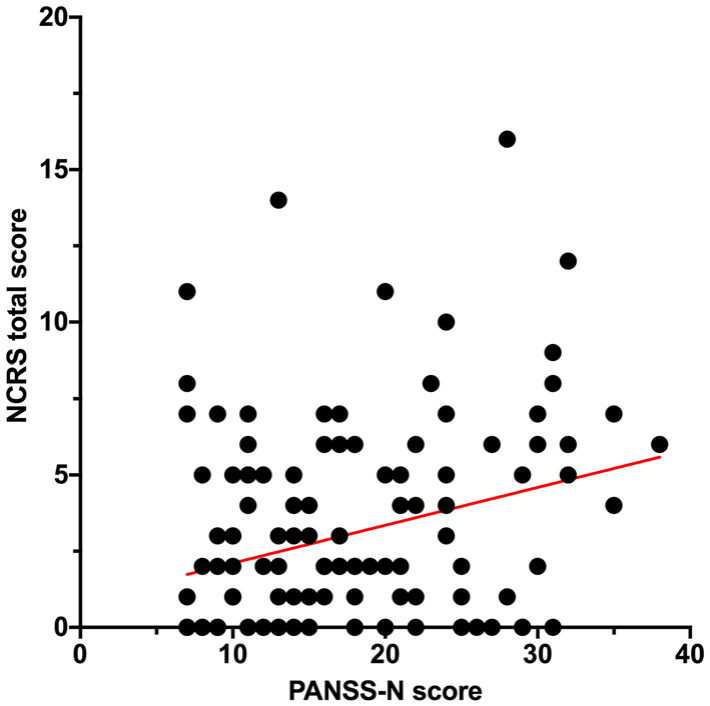
Scatter plot of linear regression analysis of Northoff Catatonia Rating Scale (NCRS) total scores and Positive and Negative Syndrome Scale (PANSS) negative score in the whole study sample (*n* = 131)

In the whole sample (*n* = 131), there was no significant association between SAS and NCRS total scores (*r*: 0.148, *p* = 0.097) or psychomotor slowing (BPRS item #13) and SAS (*r*: 0.014, *p* = 0.88) and NCRS total scores (*r*: 0.013, *p* = 0.882) after controlling for age, gender, OLZ and PANSS-N scores (Fig. 2). We found a significant correlation between PANSS-N and SAS total (*r*: 0.209, *p* = 0.018), NCRS total (*r*: 0.276, *p* = 0.002) and BPRS item #13 (*r*: 0.595, *p* < 0.001) scores after controlling for age, gender, and OLZ (Figs. 2 and 3). Only the relationship between PANSS-N scores and NCRS total and BPRS item #13 scores survived the Bonferroni correction for multiple testing (*p* = 0.05/4; *p* = 0.012) (Fig. 3). Except for glabella sign (*p* = 0.002), there was no significant association between OLZ and SAS or AIMS scores (according to Pearson correlation; Fig. 2). After the exclusion of 7 schizoaffective and 5 schizotypal disorder patients, the analyses confirmed most of our findings (see supplementary material for results).

### Clustering of SSD patients

Results from the hierarchical cluster analysis showed that the 131 SSD patients are optimally clustered into three subgroups. The first cluster included 106 SSD patients (80.9%), the second cluster 16 SSD patients (12.2%), and the third cluster nine SSD patients (6.8%) (Table 2). The results of silhouette analysis to validate our visual choice of three clusters supported this choice (s. supplementary materials). Results of linear DFA with a split-half of the sample to test reliability were virtually unchanged from the whole data-set (s. supplementary materials).

**Table 2:** Descriptive statistics of the three sensorimotor clusters and group-wise comparison across demographic, functional, cognitive and psychopathological variables

	Cluster						
	Moderate (n = 106)	Hyperkinetic (n = 9)	Hypokinetic (n = 16)	*Df*	*F*-value/ X^2^	*p*-value	Post-hoc pair-wise *t*-test
Diagnosis distribution	SZ = 96; SZA = 5, SZT = 5	SZ = 9	SZ = 14; SZA = 2	–	–	–	–
Age (years)*	36.56 (10.63)	43.11 (14.97)	47.25 (12.03)	2	7.329	** < 0.001**	Hyperkinetic vs. Hypokinetic *n.s* *Hyperkinetic vs. Moderate* n.s *Hypokinetic vs. Moderate **p* = 0.0014*
Sex°(m/f)	61/45	7/2	5/11	2	5.80	0.055	–
Education*	13.18 (2.95)	13.0 (1.32)	12.19 (2.81)	2	0.839	0.434	–
OLZe*	17.16 (10.65)	22.03 (12.54)	17.51 (8.30)	2	0.88	0.414	–
DOI (years)*	8.67 (9.86)	17.67 (10.94)	19.38 (13.19)	2	9.625	** < 0.001**	Hyperkinetic vs. Hypokinetic *n.s* *Hyperkinetic vs. Moderate ****p* = *0.04*** *Hypokinetic vs. Moderate **p* < 0.001*
GAF^#^	70.65 (17.32)	61.11 (14.53)	65.62 (15.48)	2	1.814	0.1674	–
DSST^#^	52.74 (27.51)	43.89 (17.68)	35.12 (14.51)	2	1.05	0.35	–
TMT-B^#^	98.35 (52.66)	162.22 (70.85)	188.44 (85.52)	2	10.7174	** < 0.001**	Hyperkinetic vs. Hypokinetic n.s *Hyperkinetic vs. Moderate **p* < 0.001* *Hypokinetic vs. Moderate **p* < 0.001*
PANSS^#^	65.8 (21.46)	78.00 (13.61)	69.62 (20.14)	2	1.2451	0.2915	–

Data are given as mean (SD)

*SZ* schizophrenia patients, *SZA* schizoaffective patients, *SZT* schizotypal disorder patients, *DOI* Duration of Illness, *GAF* Global Assessment of Functioning, *DSST* Digit Symbol Substitution Test, *TMT-B* Trail-Making-Test B, completion time given in seconds, *PANSS* Positive and Negative Syndrome Scale, *Df* degrees of freedom, *m/f* male/female, *n.s.* not significant. *X*^2^  Chi-squared

^*^The *F*- and *p*-values were obtained using analysis of variance (ANOVA). *SD* standard deviation. Significant *p*-values after ANOVA or post-hoc pair-wise *t*-test are indicated in bold. Significant *p*-values that survived the Bonferroni correction (*p* = 0.005) are highlighted with asterisk

^°^*X*^2^ values were obtained using Chi-Square test

^#^The *F*- and *p*-values were obtained using two-way analysis of covariance (ANCOVA) adjusted for age, gender, education and medication. SD = standard deviation. Significant *p*-values after ANCOVA or post-hoc pair-wise *t*-test are indicated in bold. Significant *p*-values that survived the Bonferroni correction (*p* = 0.005) are highlighted with asterisk

### Comparison between sensorimotor clusters on sensorimotor functioning

**Fig. 4:**
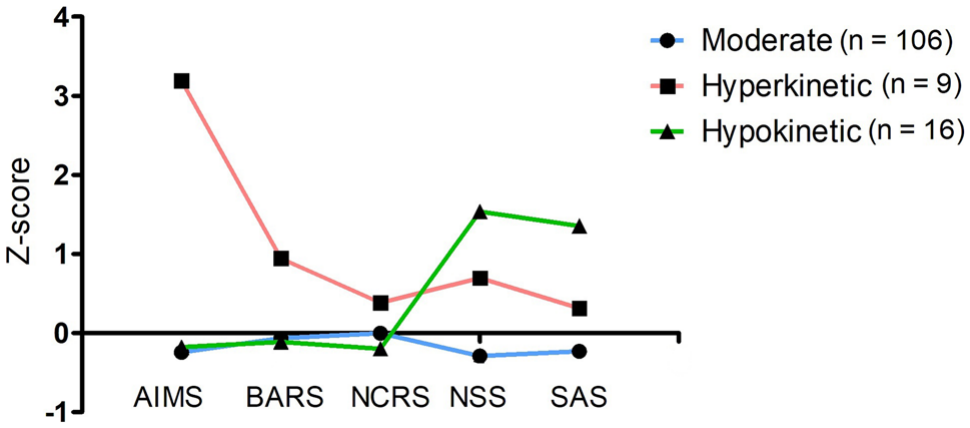
Z-scores across five distinct sensorimotor categories for each of the three sensorimotor clusters. Distinct patterns of sensorimotor abnormalities are demonstrated for the three clusters. *AIMS* Abnormal Involuntary Movement Scale, *BARS* = Barnes Akathisia Rating Scale, *NCRS*  Northoff Catatonia Rating Scale, *NSS* Neurological Soft Signs Scale, *SAS* Simpson Angus Scale

**Fig. 5:**
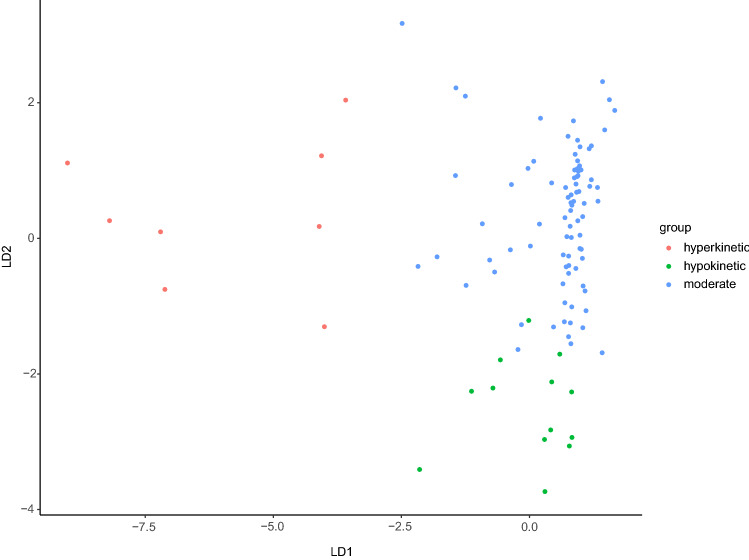
Agglomeration of SSD subjects using discriminant function analysis. The figure demonstrates the agglomeration of subjects using the three clusters emerged from the hierarchical cluster analysis. Shown are the moderate, hyperkinetic and hypokinetic groups

Visual comparison of standardized Z-scores showed that subjects in the first cluster exhibit below average sensorimotor abnormalities in all five sensorimotor categories, thus this cluster was labeled “moderate” (Fig. 4). Subjects in the second cluster presented high NSS and SAS as well as below average Z-scores in the remaining sensorimotor categories, resulting in the label “hypokinetic” (Fig. 4). Subjects in the third cluster showed predominant elevations in AIMS and BARS. While Z-scores in the remaining sensorimotor categories remained elevated, they were inferior to those in the hypokinetic cluster. This third cluster was labeled “hyperkinetic” (Fig. 4). The DFA results showed two discriminant functions delineating 76.76% and 23.23% of the variance, respectively. The model accuracy in predicting subjects grouping was 0.92. The strongest negative coefficient in function 1 (LD 1) was -1.96 (AIMS), the strongest positive coefficient was 0.25 (NCRS). Subjects grouping into the sensorimotor clusters are presented in Fig. 5.

### Demographic, functional, cognitive and psychopathological correlates of the three sensorimotor clusters

Detailed statistics on demographic, functional, cognitive and psychopathological variables within the three sensorimotor clusters are summarized in Table 2.

First, according to post-hoc pairwise t-test, executive functioning (TMT-B) appears to be more impaired in hyperkinetic and hypokinetic cluster, respectively, when compared to moderate cluster. Second, according to post-hoc pairwise *t*-test, DOI appears to be longer in hyperkinetic and hypokinetic cluster, respectively, when compared to moderate cluster. Other analyses didn’t show any differences between the three clusters in other variables (Table 2).

Second, multiple linear regression analyses showed significant (Bonferroni-corrected *p* = 0.005) relationships between CSM score of the moderate cluster and GAF and PANSS total scores (Table  3).

**Table 3:** Results of linear regression between demographic, functional, cognitive and psychopathological variables and composite sensorimotor (CSM) score

Hyperkinetic cluster (*n* = 9)	Adjusted *R*^2^	*F*-statistic	*p*-value
CSM score ~ PANSS total	− 0.1104	*F*(1,7) = 0.2049	0.664
CSM score ~ TMT-B	− 0.1335	*F*(1,7) = 0.05789	0.816
CSM score ~ DSST	− 0.09781	*F*(1,7) = 0.2872	0.609
CSM score ~ GAF	0.2967	*F*(1,7) = 4.375	0.074
CSM score ~ Age	− 0.1171	*F*(1,7) = 0.1613	0.7
CSM score ~ Sex	− 0.118	*F*(1,7) = 0.15556	0.705
CSM score ~ Education	− 0.09937	*F*(1,7) = 0.2769	0.61
CSM score ~ OLZ	− 0.08767	*F*(1,7) = 0.3552	0.57
CSM score ~ DOI	− 0.01075	*F*(1,7) = 0.9149	0.37
Hypokinetic cluster (*n* = 16)			
CSM score ~ PANSS total	0.02869	*F*(1,14) = 1.443	0.249
CSM score ~ TMT-B	0.1091	*F*(1,14) = 2.837	0.114
CSM score ~ DSST	0.02834	*F*(1,14) = 1.438	0.25
CSM score ~ GAF	0.174	*F*(1,14) = 4.1459	0.067
CSM score ~ Age	− 0.05137	*F*(1,14) = 0.2671	0.613
CSM score ~ Sex	− 0.06653	*F*(1,14) = 0.06424	0.803
CSM score ~ Education	− 0.05625	*F*(1,14) = 0.2012	0.66
CSM score ~ OLZ	0.03138	*F*(1,14) = 1.486	0.243
CSM score ~ DOI	0.003798	*F*(1,14) = 1.057	0.321
Moderate cluster (*n* = 106)			
CSM score ~ PANSS total	0.1828	*F*(1,104) = 24.49	< 0.001*
CSM score ~ TMT-B	0.009392	*F*(1,104) = 1.996	0.160
CSM score ~ DSST	0.03395	*F*(1,104) = 4.69	**0.032**
CSM score ~ GAF	0.1946	*F*(1,104) = 26.37	< 0.001*
CSM score ~ Age	− 0.0951	*F*(1,104) = 0.01089	0.917
CSM score ~ Sex	− 0.009133	*F*(1,104) = 0.04971	0.824
CSM score ~ Education	− 0.007308	*F*(1,104) = 0.2382	0.626
CSM score ~ OLZ	0.05823	*F*(1,104) = 7.492	**0.007**
CSM score ~ DOI	0.01473	*F*(1,104) = 2.57	0.112

Data represent the results of linear regression between composite sensorimotor scores (calculated as mean from z-standardized AIMS, BARS, NCRS, NSS and SAS scores) and clinical correlates. The analyses were separately conducted in each cluster. Significant *p*-values that survived the Bonferroni correction (*p* = 0.005) are highlighted with asterisk

*PANSS* Positive and Negative Syndrome Scale, *AIMS* Abnormal involuntary movement scale, *BARS* Barnes Akathisia Rating Scale, *NCRS* Northoff Catatonia Rating Scale, *NSS* Neurological Soft Signs Scale, *SAS* Simpson-Angus-Scale, *TMT-B* Trail Making Test B, *DSST* Digit Symbol Substitution Test, *OLZ* Olanzapine equivalents, *DOI* Duration of Illness. Significant *p*-values are highlighted in bold. Significant *p*-values that survived the Bonferroni correction (*p* = 0.005) are highlighted with asterisk

## Discussion

This study comprehensively explored sensorimotor abnormalities in SSD, particularly symptom interrelationships as well as associations between sensorimotor symptoms, psychopathology and cognition. Four main findings emerged: (1) NSS were clearly the most frequently observed sensorimotor abnormality in our sample, followed by parkinsonism, akathisia, catatonia and TD. (2) Sensorimotor abnormalities tended to overlap, with the overlap between NSS and parkinsonism being the most frequent, followed by NSS and akathisia, followed by NSS and catatonia. (3) NCRS total scores were associated with PANSS-N scores in SSD patients. (4) Hierarchical cluster analysis and DFA demonstrated three sensorimotor clusters (“moderate”, “hyperkinetic” and “hypokinetic”) and the hyperkinetic and hypokinetic groups differed significantly in their TMT-B performance compared with the moderate group.

Ad (1) First of all, our results underline the clinical relevance of sensorimotor abnormalities in SSD. In our sample, 128 of 131 patients (97.7%) satisfied criteria for at least one sensorimotor abnormality. The prevalence of NSS in our study is comparable to other reports (20–97% in first-episode patients and 60% in patients treated with antipsychotics) [1], yet rather at the upper end of the spectrum. This may in part be due to differences in rating scale selection and differing cut-offs defining the presence of NSS [1]. We chose a rather low NSS cut-off, possibly contributing to the high prevalence of NSS. The prevalence of parkinsonism, akathisia and catatonia is rather similar within our results. In comparison with the study conducted by Peralta and colleagues [14], our prevalence rates are slightly higher, which could be accounted for by the different sample characteristics (patients were older, had a longer duration of illness and a longer exposure to antipsychotic treatment) in our study. The prevalence of akathisia in our study is in line with an earlier report [44], yet distinctly higher in comparison with Peralta and colleagues [14]. Interestingly, in spite of our sample comprising older patients with longer duration of illness and more antipsychotic exposure, the prevalence of TD is somewhat lower in our study compared to the study by Peralta and colleagues [14]. We employed the same criteria to define TD, so this result could lend further support to the hypothesis of a complex relationship of sensorimotor abnormalities and antipsychotics, sometimes exacerbating and sometimes improving sensorimotor abnormalities [7, 14].

Ad (2) The overlaps of sensorimotor abnormalities found in our sample were larger than in the study by Peralta and colleagues [14], probably to some extent because of the inclusion of NSS and its high prevalence (96%) in our comparably older sample with longer antipsychotic exposure. Interestingly, we found a significant overlap not only between NSS and parkinsonism and between NSS and catatonia, but also between NSS and akathisia, which to the best of our knowledge has not been reported yet. In fact, there are several reports that correlated NSS and akathisia, with negative results [45, 46]. A possible explanation for these studies´ failure to detect an overlap might be their lower prevalence of akathisia, reducing pre-test probability and thus statistical power [14]. We do not feel that our higher prevalence rates of akathisia could be due to the so called “pseudoakathisia”, as sometimes discussed in the literature [14], yet, at the same time, we cannot fully exclude this possibility [47, 48].

The phenomenon of co-occurrence of sensorimotor abnormalities has been discussed in detail by Walther and colleagues [7]. We agree with these authors in their argument that until today, it is difficult to determine whether co-occurring sensorimotor abnormalities may be due to lack of conceptual clarity, a strong intercorrelation, or a common neuronal basis [7]. The difficulty of conceptual clarity can be illustrated when considering that reduced level of motion can be described as stupor, akinesia, retardation or bradykinesia. Similarly, rigidity has been considered as a sign of parkinsonism as well as catatonia. NSS scales often include items present in scales for parkinsonism or dyskinesia, such as tremor, rigor or difficulty with balance. On the other hand, catatonia scales often include rigor. In line with this, Peralta and colleagues [45] reported significant correlations between spontaneous movement disorders and NSS (*r* = 0.36, *p* < 0.001) in schizophrenia. To examine the clinical correlates of reduced level of movement, we assessed the association between psychomotor slowing (BPRS item #13) and SAS as well as NCRS. The lack of association in our data suggests that psychomotor slowing and parkinsonism as well as catatonia might be clinically specific sensorimotor phenomena. From a neurobiological perspective, motor, behavioral and affective symptoms of catatonia could be accounted for by distinct dysfunction in orbitofrontal-prefrontal/parietal cortical connectivity ("horizontal modulation") and basal ganglia („vertical modulation") [49–51]. Concerning parkinsonism, a vast majority of studies emphasized that motor symptoms could be attributed to altered "bottom-up modulation" from basal ganglia to cortex [15, 50, 52, 53]. Based on this evidence, it can be said that these phenomena also have different underlying pathomechanism.

Ad (3) Our finding that NCRS total scores are associated with PANSS-N scores in SSD patients suggests that catatonic signs are an intrinsic component of SSD pathophysiology. This finding is well in line with previous studies [14, 54–56]. This relationship reflects the daily clinical practice, because it is not always easy to distinguish between the different sensorimotor categories. Furthermore, negative symptoms may also modulate the sensorimotor domain, especially when patients suffer from akinesia and psychomotor slowing. Last but not least, this result lends further evidence to the proposed use of catatonic symptoms as a severity marker and to predict treatment response [14].

Ad (4) Using hierarchical clustering analysis and DFA, we were able to show for the first time that sensorimotor categories can be classified into three different, but clinically plausible clusters. In particular, the hyperkinetic cluster is characterized by AIMS and akathisia. The hypokinetic cluster is characterized by NSS and parkinsonism. The moderate cluster, with the highest number of patients, is characterized by all five sensorimotor categories with moderate severity of symptoms. The three clusters are clinically plausible and may help to define subtypes of the heterogenous SSD syndrome, benefitting future diagnostic efforts. In addition, our results support the phenomenon of co-occurrence of sensorimotor abnormalities described by Walther and colleagues [7] and are also in line with a recent actigraphy study by Pieters and colleagues [57] that showed a significant relationship between parkinsonism and low physical activity and between akathisia and higher physical activity.

Ad (5) We explored the relationship between the identified three sensorimotor clusters and their psychopathological and neurocognitive correlates. We chose GAF, TMT-B and PANSS as correlates since they inform about general, cognitive, psychomotor and psychopathological aspects of SSD, respectively. Interestingly, the three clusters differed in severity of the executive functioning deficits according to TMT-B. This is also in line with previous evidence that showed more pronounced hypokinetic sensorimotor abnormalities (NSS and parkinsonism) to be related to executive functioning deficits in SSD patients [58, 59]. Furthermore, our results support a specific relationship between the sensorimotor and cognitive domain, as showed previously by Wolf and colleagues [60]. In addition, TMT-A and -B as well as DSST are popular measures of cognitive functioning, especially processing speed, cognitive flexibility and the so-called psychomotor slowing. Following this line of thought, a broader definitions of this term may include sensorimotor abnormalities such as NSS, catatonia and parkinsonism [61]. In line with this, the cognitive (“psycho”) and motor sub-processes comprising psychomotor slowing as described by Osborne et al. [61] may overlap with sub-processes of sensorimotor abnormalities. Psychomotor slowing sub-processes may require visuo-motor transformations including initial perception of relevant stimuli, keeping these in working memory until a decision according to intended actions is determined, followed by a subsequent response [61]. To prove the validity of the identified clusters and to clarify the relationship between different domains, further neuroimaging and longitudinal studies are needed. Such studies should also try to link neurobiological markers (such as brain imaging or epigenetic markers) with different and clinically plausible sensorimotor clusters.

### Limitations

Despite the apparent advantages of the study (large sample size, close to clinical reality, comprehensive examination of sensorimotor abnormalities), there are some limitations: first, the cross-sectional design does not allow inferences about symptom stability or dynamics over time. Second, the employed sensorimotor scales use partly overlapping items and despite sufficient inter-rater reliability, sensorimotor assessment may benefit of complementary instrumental and ecological momentary assessments in future research. Third, we could not record the entire history of antipsychotic medication in the present patient sample, because central patient registries are not available at the national or regional level. Therefore, antipsychotic treatment may still be considered as a potential confounder of sensorimotor assessment and the current daily dosage may not be a reliable reflection of the life-long cumulative effects of antipsychotics on sensorimotor system in the present patient sample. Although at present it is not possible to distinguish genuine from antipsychotic-induced sensorimotor abnormalities in patients treated with antipsychotic agents, it is worth noting that in this study, only four patients received first-generation antipsychotic and the vast majority of patients were treated with second-generation antipsychotics. Except for glabella sign, there was no significant association between OLZ and SAS or AIMS scores. However, it is unclear whether glabella sign can be modulated by medication because it belongs to the frontal release signs (also known as primitive reflexes), which disappear in the course of further brain development [5]. In SSD patients, frontal release signs might get disinhibited by illness immanent alterations of the fronto-parietal areas [6]. Therefore, it is possible that if medication may not show sufficient effect, disinhibition and development of glabella signs will occur again. In this particular case, glabella sign is not a side effect, but possibly indicative of an insufficient effect of drug treatment. Similarly, recent evidence showed that antipsychotic drug dose had no effect on the severity of antipsychotic-induced sensorimotor abnormalities and the prevalence of antipsychotic-associated estimates of sensorimotor abnormalities was not influenced by treatment duration [64, 65]. Finally, sensorimotor abnormalities might result from an exposure to not only dopamine receptor-blocking agents (e.g. first- and second-generation antipsychotics), but also other agents such as tricyclic antidepressants, lithium, serotonin reuptake or serotonin norepinephrine reuptake inhibitors, calcium channel blockers, antiemetics, and other medications used for gastrointestinal disorders), respectively [52, 53, 66, 67]. Therefore, it is important to perform regular screenings for sensorimotor abnormalities in patients with polypharmacy. Nonetheless, future studies need to examine either antipsychotic-naive patients or sufficiently large groups of patients on different types of antipsychotic agents [52, 53, 67]. Finally, we examined a total of five different sensorimotor categories. From a clinical perspective, it can be claimed that these categories have different characteristics not only in a cross-sectional examination, but also in a longitudinally. On one side, catatonic symptoms (e.g. periodic catatonia [68]), akathisia, and to some extent also NSS may fluctuate throughout the day or week with relapsing and fully remitting course. In particular, excitement, waxy flexibility and immobility/stupor are catatonic symptoms which best describe the three different catatonic subtypes characterized by increased, abnormal and decreased psychomotor activity [69]. On the other side, parkinsonism and TD tend to be more temporally stable sensorimotor abnormalities. Although TD can also have a waxing and waning course, it is a chronic syndrome that can persist for years or decades even though the causative drug has been discontinued. However, the available clinical rating scales are only able to capture the static characteristics of these categories and hence, the majority of the sensorimotor scales did not report test–retest reliability (for a review see [70]). Since sensorimotor abnormalities might show both qualitative and quantitative fluctuations over time, they need to be assessed longitudinally in both research and clinical setting. Therefore, future studies should assess all categories of sensorimotor abnormalities on both micro- (e.g. ecological momentary and day-to-day assessments) and macro-levels (weeks and months) [71].

## Conclusion

The vast majority of SSD patients were shown to exhibit at least one sensorimotor abnormality. The spectrum ranges from subtle sensorimotor abnormalities in terms of NSS to catatonia and/or more pronounced TD. Overall, this study emphasizes the crucial role of sensorimotor abnormalities and underline its potential as research target to improve diagnosis and treatment efforts in SSD.

## Supplementary Information

Below is the link to the electronic supplementary material.

Supplementary file1 (DOCX 3990 kb)

## Data Availability

All original data are on record and accessible to inspection, but are not available publicly based on local and national data protection regulations.

## Code availability

All software used in the analyses is based on publicly available code.

## References

[1] Bachmann S, Schröder J (2017). Neurological soft signs in schizophrenia: an update on the state- versus trait-perspective. Front Psych.

[2] Hirjak D, Thomann PA, Kubera KM, Wolf ND, Sambataro F, Wolf RC (2015). Motor dysfunction within the schizophrenia-spectrum: a dimensional step towards an underappreciated domain. Schizophr Res.

[3] Northoff G, Hirjak D (2020) All roads lead to the motor cortex: Psychomotor mechanisms and their biochemical modulation in psychiatric disorders. 10.1038/s41380-020-0814-5 32555423

[4] Walther S, van Harten PN, Waddington JL, Cuesta MJ, Peralta V, Dupin L, Foucher JR, Sambataro F, Morrens M, Kubera KM, Pieters LE, Stegmayer K, Strik W, Wolf RC, Hirjak D (2020). Movement disorder and sensorimotor abnormalities in schizophrenia and other psychoses—European consensus on assessment and perspectives. Euro Neuropsychopharmacol.

[5] Sanislow CA, Ferrante M, Pacheco J, Rudorfer MV, Morris SE (2019). Advancing translational research using NIMH research domain criteria and computational methods. Neuron.

[6] Peralta V, Cuesta MJ (2017). Motor abnormalities: from neurodevelopmental to neurodegenerative through "functional" (neuro)psychiatric disorders. Schizophr Bull.

[7] Walther S, Strik W (2012). Motor symptoms and schizophrenia. Neuropsychobiology.

[8] Chan RC, Xu T, Heinrichs RW, Yu Y, Gong QY (2010). Neurological soft signs in non-psychotic first-degree relatives of patients with schizophrenia: a systematic review and meta-analysis. Neurosci Biobehav Rev.

[9] Hirjak D, Kubera KM, Thomann PA, Wolf RC (2018). Motor dysfunction as an intermediate phenotype across schizophrenia and other psychotic disorders: progress and perspectives. Schizophr Res.

[10] Hirjak D, Meyer-Lindenberg A, Fritze S, Sambataro F, Kubera KM, Wolf RC (2018). Motor dysfunction as research domain across bipolar, obsessive-compulsive and neurodevelopmental disorders. Neurosci Biobehav Rev.

[11] Hirjak D, Meyer-Lindenberg A, Kubera KM, Thomann PA, Wolf RC (2018). Motor dysfunction as research domain in the period preceding manifest schizophrenia: a systematic review. Neurosci Biobehav Rev.

[12] Zhao Q, Li Z, Huang J, Yan C, Dazzan P, Pantelis C, Cheung EF, Lui SS, Chan RC (2014). Neurological soft signs are not "soft" in brain structure and functional networks: evidence from ale meta-analysis. Schizophr Bull.

[13] Andreasen NC (2000). Schizophrenia: the fundamental questions. Brain Res Brain Res Rev.

[14] Peralta V, Cuesta MJ (2011). Neuromotor abnormalities in neuroleptic-naive psychotic patients: antecedents, clinical correlates, and prediction of treatment response. Compr Psychiatry.

[15] Waddington JL (2020). Psychosis in Parkinson's disease and parkinsonism in antipsychotic-naive schizophrenia spectrum psychosis: clinical, nosological and pathobiological challenges. Acta Pharmacol Sin.

[16] Sambataro F, Fritze S, Rashidi M, Topor CE, Kubera KM, Wolf RC, Hirjak D (2020). Moving forward: distinct sensorimotor abnormalities predict clinical outcome after 6 months in patients with schizophrenia. Euro Neuropsychopharmacol.

[17] Janno S, Holi M, Tuisku K, Wahlbeck K (2004). Prevalence of neuroleptic-induced movement disorders in chronic schizophrenia inpatients. Am J Psychiatry.

[18] Hirjak D, Meyer-Lindenberg A, Sambataro F, Fritze S, Kukovic J, Kubera KM, Wolf RC (2021) Progress in sensorimotor neuroscience of schizophrenia spectrum disorders: Lessons learned and future directions. Progress in neuro-psychopharmacology & biological psychiatry 111:110370 10.1016/j.pnpbp.2021.110370 34087392

[19] Hirjak D, Kubera KM, Northoff G, Fritze S, Bertolino AL, Topor CE, Schmitgen MM, Wolf RC (2019). Cortical contributions to distinct symptom dimensions of catatonia. Schizophr Bull.

[20] Hirjak D, Rashidi M, Kubera KM, Northoff G, Fritze S, Schmitgen MM, Sambataro F, Calhoun VD, Wolf RC (2020). Multimodal magnetic resonance imaging data fusion reveals distinct patterns of abnormal brain structure and function in catatonia. Schizophr Bull.

[21] Oldfield RC (1971). The assessment and analysis of handedness: The Edinburgh inventory. Neuropsychologia.

[22] Sass H., Wittchen H.U., Zaudig M., I. H (2003) Diagnostisches und statistisches manual psychischer störungen dsm-iv-tr: Textrevision. Hogrefe Verlag; Auflage: 1 (1. Januar 2003)

[23] Hirjak D, Kubera KM, Northoff G, Fritze S, Bertolino AL, Topor CE, Schmitgen MM, Wolf RC (2019) Cortical contributions to distinct symptom dimensions of catatonia. Schizophr Bull 10.1093/schbul/sby192 PMC6811823 30753720

[24] Hirjak D, Rashidi M, Kubera KM, Northoff G, Fritze S, Schmitgen MM, Sambataro F, Calhoun VD, Wolf RC (2019) Multimodal magnetic resonance imaging data fusion reveals distinct patterns of abnormal brain structure and function in catatonia. Schizophrenia bulletin 10.1093/schbul/sbz042 PMC6942158 31174212

[25] Leucht S, Samara M, Heres S, Patel MX, Furukawa T, Cipriani A, Geddes J, Davis JM (2015). Dose equivalents for second-generation antipsychotic drugs: the classical mean dose method. Schizophr Bull.

[26] Kay SR, Fiszbein A, Opler LA (1987). The positive and negative syndrome scale (panss) for schizophrenia. Schizophr Bull.

[27] Kay SR (1990). Positive-negative symptom assessment in schizophrenia: psychometric issues and scale comparison. Psychiatr Q.

[28] Andersen J, Larsen JK, Kørner A, Nielsen BM, Schultz V, Behnke K, Bjørum N (1986) The brief psychiatric rating scale: Schizophrenia, reliability and validity studies. Nordisk Psykiatrisk Tidsskrift 40:2.:135–138

[29] Overall JE, Gorham DR (1962) The brief psychiatric rating scale (bprs). Psychological reports 10:799–812 10:799–812.

[30] Berk M, Ng F, Dodd S, Callaly T, Campbell S, Bernardo M, Trauer T (2008). The validity of the cgi severity and improvement scales as measures of clinical effectiveness suitable for routine clinical use. J Eval Clin Pract.

[31] Guy W (1976) Assessment manual for psychopharmacology. Rockville/ Ts, NIH Psychopharmacology Research:76–338.

[32] Grootenboer EM, Giltay EJ, van der Lem R, van Veen T, van der Wee NJ, Zitman FG (2012). Reliability and validity of the global assessment of functioning scale in clinical outpatients with depressive disorders. J Eval Clin Pract.

[33] DSM-III.R. DKuDddusMpSr (1989) Gaf-skala: Global assessment of functioning scale. In:Beltz, Weinheim, Basel.

[34] Hurford IM, Marder SR, Keefe RS, Reise SP, Bilder RM (2011). A brief cognitive assessment tool for schizophrenia: construction of a tool for clinicians. Schizophr Bull.

[35] Bachmann S, Bottmer C, Schroder J (2005). Neurological soft signs in first-episode schizophrenia: a follow-up study. Am J Psychiatry.

[36] Schroder J, Niethammer R, Geider FJ, Reitz C, Binkert M, Jauss M, Sauer H (1991). Neurological soft signs in schizophrenia. Schizophr Res.

[37] Simpson GM, Angus JW (1970). A rating scale for extrapyramidal side effects. Acta Psychiatr Scand Suppl.

[38] Hirjak D, Thomann PA, Northoff G, Kubera KM, Wolf RC (2016) Ncr-skala - deutsche version der northoff catatonia rating scale (ncrs-dv) - ein validiertes messinstrument zur erfassung katatoner symptome. Der Nervenarzt:(im Druck) 10.1007/s00115-016-0136-7 27325247

[39] Barnes TR (1989). A rating scale for drug-induced akathisia. Br J Psychiatry.

[40] Barnes TR (2003). The Barnes akathisia rating scale–revisited. J Psychopharmacol.

[41] Guy E (1976) Abnormal involuntary movement scale., Rockwille, MD

[42] Burdick KE, Russo M, Frangou S, Mahon K, Braga RJ, Shanahan M, Malhotra AK (2014). Empirical evidence for discrete neurocognitive subgroups in bipolar disorder: clinical implications. Psychol Med.

[43] Aranganayagi S, Thangavel K (2007) Clustering categorical data using silhouette coefficient as a relocating measure. In: International conference on computational intelligence and multimedia applications (ICCIMA 2007). IEEE, p 13–17

[44] Halstead SM, Barnes TR, Speller JC (1994). Akathisia: prevalence and associated dysphoria in an in-patient population with chronic schizophrenia. Br J Psychiatry.

[45] Peralta V, Moreno-Izco L, Sanchez-Torres A, García de Jalón E, Campos MS, Cuesta MJ (2014). Characterization of the deficit syndrome in drug-naive schizophrenia patients: the role of spontaneous movement disorders and neurological soft signs. Schizophr Bull.

[46] Jahn T, Hubmann W, Karr M, Mohr F, Schlenker R, Heidenreich T, Cohen R, Schröder J (2006). Motoric neurological soft signs and psychopathological symptoms in schizophrenic psychoses. Psychiatry Res.

[47] McCreadie RG, Thara R, Kamath S, Padmavathy R, Latha S, Mathrubootham N, Menon MS (1996). Abnormal movements in never-medicated Indian patients with schizophrenia. Br J Psychiatry.

[48] Stubbs JH, Halstead SM (2000). Pseudoakathisia: a review and two case reports. Compr Psychiatry.

[49] Hirjak D, Kubera KM, Wolf RC, Northoff G (2020). Going back to Kahlbaum's psychomotor (and gabaergic) origins: Is catatonia more than just a motor and dopaminergic syndrome?. Schizophr Bull.

[50] Northoff G (2002) What catatonia can tell us about "top-down modulation": A neuropsychiatric hypothesis. The Behavioral and brain sciences 25:555–577; discussion 578–604 10.1017/s0140525x02000109 12958742

[51] Northoff G (2002) Catatonia and neuroleptic malignant syndrome: Psychopathology and pathophysiology. Journal of neural transmission (Vienna, Austria : 1996) 109:1453–1467 10.1007/s00702-002-0762-z 12486486

[52] Wasserthal J, Maier-Hein KH, Neher PF, Wolf RC, Northoff G, Waddington JL, Kubera KM, Fritze S, Harneit A, Geiger LS, Tost H, Hirjak D (2021). White matter microstructure alterations in cortico-striatal networks are associated with parkinsonism in schizophrenia spectrum disorders. Euro Neuropsychopharmacol.

[53] Wolf RC, Rashidi M, Fritze S, Kubera KM, Northoff G, Sambataro F, Calhoun VD, Geiger LS, Tost H, Hirjak D (2020). A neural signature of parkinsonism in patients with schizophrenia spectrum disorders: a multimodal MRI study using parallel ica. Schizophr Bull.

[54] Jain A, Mitra P (2020) Catatonic schizophrenia. In: Statpearls. StatPearls Publishing Copyright © 2020, StatPearls Publishing LLC., Treasure Island (FL)

[55] Ungvari GS, Gerevich J, Takács R, Gazdag G (2018). Schizophrenia with prominent catatonic features: a selective review. Schizophr Res.

[56] Ungvari GS, Goggins W, Leung SK, Gerevich J (2007). Schizophrenia with prominent catatonic features ('catatonic schizophrenia'). Ii. Factor analysis of the catatonic syndrome. Prog Neuropsychopharmacol Biol Psychiatry.

[57] Pieters LE, Deenik J, Tenback DE, van Oort J, van Harten PN (2021). Exploring the relationship between movement disorders and physical activity in patients with schizophrenia: an actigraphy study. Schizophr Bull.

[58] Cuesta MJ, Sanchez-Torres AM, de Jalon EG, Campos MS, Ibanez B, Moreno-Izco L, Peralta V (2014). Spontaneous parkinsonism is associated with cognitive impairment in antipsychotic-naive patients with first-episode psychosis: a 6-month follow-up study. Schizophr Bull.

[59] Peralta V, Basterra V, Campos MS, de Jalon EG, Moreno-Izco L, Cuesta MJ (2012). Characterization of spontaneous parkinsonism in drug-naive patients with nonaffective psychotic disorders. Eur Arch Psychiatry Clin Neurosci.

[60] Wolf RC, Rashidi M, Schmitgen MM, Fritze S, Sambataro F, Kubera KM, Hirjak D (2021). Neurological soft signs predict auditory verbal hallucinations in patients with schizophrenia. Schizophr Bull.

[61] Osborne KJ, Walther S, Shankman SA, Mittal VA (2020) Psychomotor slowing in schizophrenia: Implications for endophenotype and biomarker development. Biomark Neuropsychiatry 2 10.1016/j.bionps.2020.100016 PMC7963400 33738459

[62] Hyde TM, Goldberg TE, Egan MF, Lener MC, Weinberger DR (2007). Frontal release signs and cognition in people with schizophrenia, their siblings and healthy controls. Br J Psychiatry.

[63] Schott JM, Rossor MN (2003). The grasp and other primitive reflexes. J Neurol Neurosurg Psychiatry.

[64] Parksepp M, Ljubajev U, Taht K, Janno S (2016). Prevalence of neuroleptic-induced movement disorders: an 8-year follow-up study in chronic schizophrenia inpatients. Nord J Psychiatry.

[65] Martino D, Karnik V, Osland S, Barnes TRE, Pringsheim TM (2018) Movement disorders associated with antipsychotic medication in people with schizophrenia: An overview of cochrane reviews and meta-analysis. Can J Psychiatry:706743718777392 10.1177/0706743718777392 PMC6299187 29758999

[66] Factor SA, Burkhard PR, Caroff S, Friedman JH, Marras C, Tinazzi M, Comella CL (2019). Recent developments in drug-induced movement disorders: a mixed picture. Lancet Neurol.

[67] Wolf RC, Kubera KM, Waddington JL, Schmitgen MM, Fritze S, Rashidi M, Thieme CE, Sambataro F, Geiger LS, Tost H, Hirjak D (2021). A neurodevelopmental signature of parkinsonism in schizophrenia. Schizophr Res.

[68] Foucher JR, de Billy C, Jeanjean LC, Obrecht A, Mainberger O, Clauss JME, Schorr B, Lupu MC, de Sousa PL, Lamy J, Noblet V, Sauleau EA, Landre L, Berna F (2020). A brain imaging-based diagnostic biomarker for periodic catatonia: preliminary evidence using a Bayesian approach. Neuropsychobiology.

[69] Wilson JE, Niu K, Nicolson SE, Levine SZ, Heckers S (2015). The diagnostic criteria and structure of catatonia. Schizophr Res.

[70] van Strien AM, Keijsers CJ, Derijks HJ, van Marum RJ (2015). Rating scales to measure side effects of antipsychotic medication: a systematic review. J Psychopharmacol.

[71] Nelson B, McGorry PD, Wichers M, Wigman JTW, Hartmann JA (2017). Moving from static to dynamic models of the onset of mental disorder: a review. JAMA Psychiat.

