# Supplementary material for: Characterizing the sensorimotor domain in schizophrenia spectrum disorders
Source: Eur Arch Psychiatry Clin Neurosci. 2021 Nov 27;272(6):1097–108. doi: 10.1007/s00406-021-01354-9 (PMC9388408; doi:10.1007/s00406-021-01354-9)
Supplement: Supplementary file 1 — Supplementary file1 (DOCX 3990 kb) [file 406_2021_1354_MOESM1_ESM.docx]

**Supplementary material:**

*Study participants*

Patients were excluded if: (i) they were <18 years or >65 years; (ii) they had a history of brain trauma or neurological disease (especially movement disorders); or (iii) they had shown alcohol/substance use disorder within 12 months before participation. Medical conditions potentially affecting central nervous system function, as well as cardiovascular or metabolic diseases in SSD patients were excluded by physical examination, laboratory control, ECG and MRI.

*Clinical assessment*

Heidelberg NSS consists of five items assessing motor coordination (MOCO) [Ozeretski’s test, diadochokinesia, pronation/supination, finger-to-thumb opposition, speech articulation], three items assessing integrative functions (IF) [station and gait, tandem walking, two-point discrimination], two items assessing complex motor tasks (COMT) [finger-to-nose test, fist-edge-palm test], four items assessing right/left and spatial orientation (RLSPO) [right/left orientation, graphesthesia, face-hand test, stereognosis], and two items assessing hard signs (HS) [arm holding test, mirror movements]. Ratings are given on a 0 (no prevalence) to 3 (marked prevalence) point scale. A sufficient internal reliability and test-retest reliability have been established previously [1, 2]. The SAS scale [3] assesses 10 items (gait, arm dropping, shoulder shaking, elbow rigidity, wrist rigidity, leg pendulousness, head dropping, glabella tap, tremor, salivation) on a scale of 0 (no prevalence) to 4 (marked prevalence) points. The NCRS [4] measures the presence and severity of motor (13 items), affective (12 items), and behavioral (15 items) catatonic symptoms on a scale of 0 (not present) to 2 points (marked presence). The BARS uses the items objective, subjective (consisting of awareness and distress related to restlessness) and global clinical assessment on a scale of 0 (absence/no distress) to 3 points (marked or severe presence). The AIMS uses the items muscles of facial expression, lips and perioral, jaw, tongue, upper extremities, lower extremities, trunk, movements, severity of abnormal movements, incapacitation due to abnormal movements and patient´s awareness of abnormal movements on a scale of 0 (absence) to 4 points (severe).

The threshold value for NSS was a NSS total score of >7 (according to a mean value in healthy individuals [5, 6]. A cutoff score of >3 on the total SAS score was employed to define parkinsonism based on previous studies addressing parkinsonism [7, 8]. The presence of catatonia was defined by a cut-off score of ≥1 on each of the three subscales and NCRS total score ≥3 [9, 10]. The threshold value for akathisia was a BARS global score of 2 or more [11]. Patients with tardive dyskinesia were defined by the AIMS according to Schooler-Kane criteria [12, 13], which require at least moderate dyskinetic movements in one body area or mild dyskinetic movements in two body areas [11].

**Supplementary table 1.** Number of patients with overlapping sensorimotor dysfunction.

| Number of patients overlapping in two sensorimotor scores | | | | |
| --- | --- | --- | --- | --- |
| AIMS & BARS | AIMS & NCRS | AIMS & NSS | AIMS & SAS | NSS & SAS |
| **5** | **4** | **8** | **3** | **43** |
| BARS & NCRS | BARS & NSS | BARS & SAS | NCRS & NSS | NCRS & SAS |
| **12** | **35** | **10** | **31** | **14** |
| Number of patients overlapping in three sensorimotor scores | | | | |
| AIMS & BARS & NCRS | AIMS & BARS & NSS | AIMS & BARS & SAS | AIMS & NCRS & NSS | AIMS & NCRS & SAS |
| **3** | **5** | **2** | **4** | **2** |
| AIMS & NSS & SAS | BARS & NCRS & NSS | BARS & NCRS & SAS | BARS & NSS & SAS | BARS & NSS & SAS |
| **3** | **12** | **5** | **10** | **10** |
| NCRS & NSS & SAS |  |  |  |  |
| **14** |  |  |  |  |
| Number of patients overlapping in four sensorimotor scores | | | | |
| AIMS & BARS & NCRS & NSS | AIMS & BARS & NCRS & SAS | BARS & NCRS & NSS & SAS | AIMS & NCRS & NSS & SAS | AIMS & BARS & NSS & SAS |
| **3** | **2** | **5** | **2** | **2** |

Data represent the number of patients satisfying criteria for more than one sensorimotor abnormality, *e.g. the number of patients satisfying* *AIMS and BARS criteria was n = 5*.

Abbreviations: AIMS=Abnormal involuntary movement scale, BARS=Barnes Akathisia Rating Scale, NCRS=Northoff Catatonia Rating Scale, NSS= Neurological Soft Signs Scale, SAS=Simpson-Angus-Scale.

**Supplementary table 2.**

| Comparison between the three sensorimotor clusters across distinct sensorimotor abnormalities employing Z-scores. Data are given as mean. | | | |
| --- | --- | --- | --- |
|  | Cluster |  |  |
|  | Moderate (n = 106) | Hyperkinetic (n = 9) | Hypokinetic (n = 16) |
|  |  |  |  |
| AIMS | -0.2442974 | 3.1904683 | -0.1761682 |
| BARS | -0.06339282 | 0.94379904 | -0.11090953 |
| NCRS | -0.002199288 | 0.382583297 | -0.200632820 |
| NSS | -0.2910914 | 0.6953631 | 1.5373391 |
| SAS | -0.2310330 | 0.3153936 | 1.3531850 |

Abbreviations: AIMS = Abnormal Involuntary Movement Scale. BARS = Barnes Akathisia Rating Scale. NCRS = Northoff Catatonia Rating Scale. NSS = Neurological Soft Signs Scale. SAS = Simpson Angus Scale.

**Supplementary figure 1.**


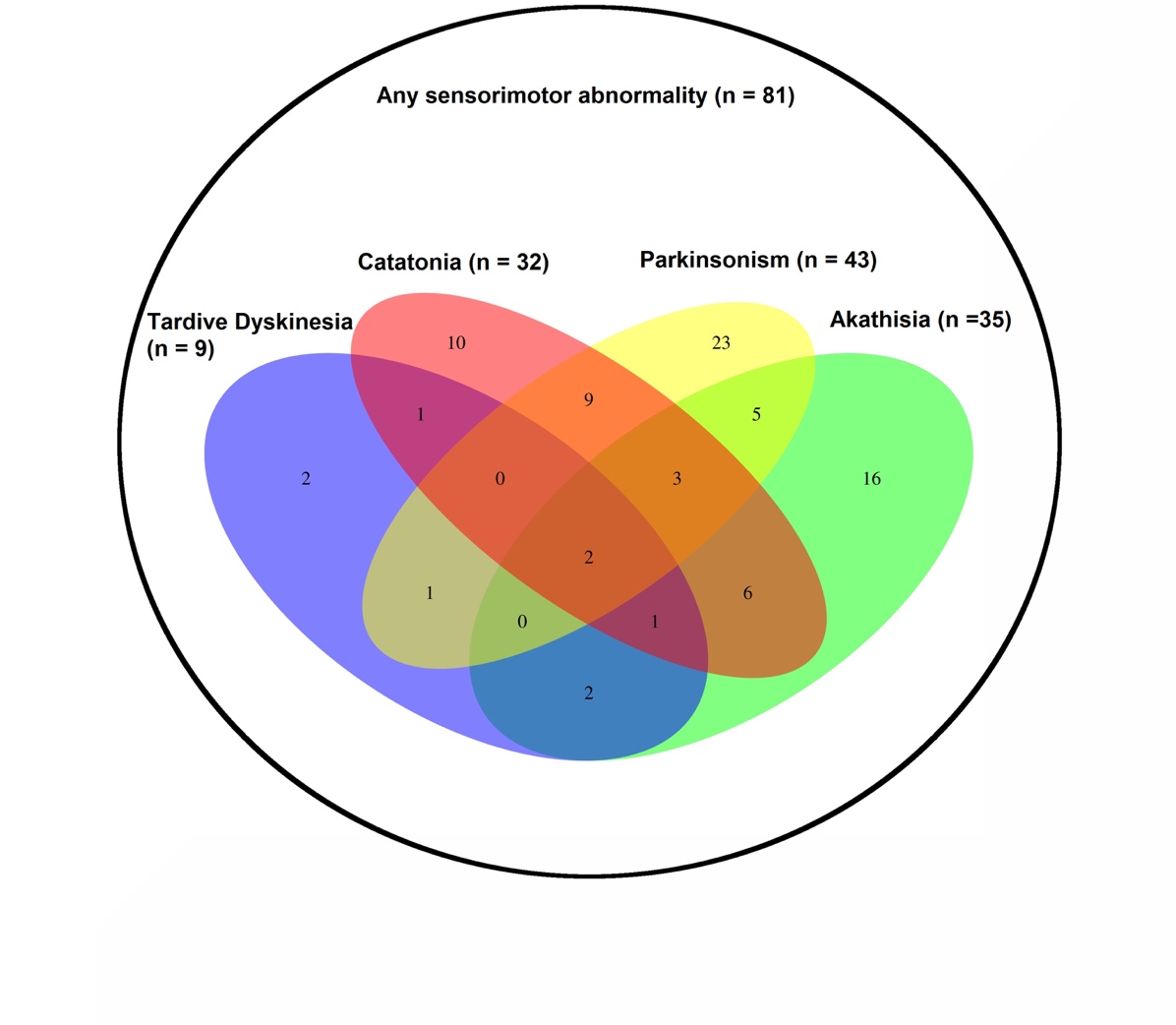


**Supplementary figure 2.**

**
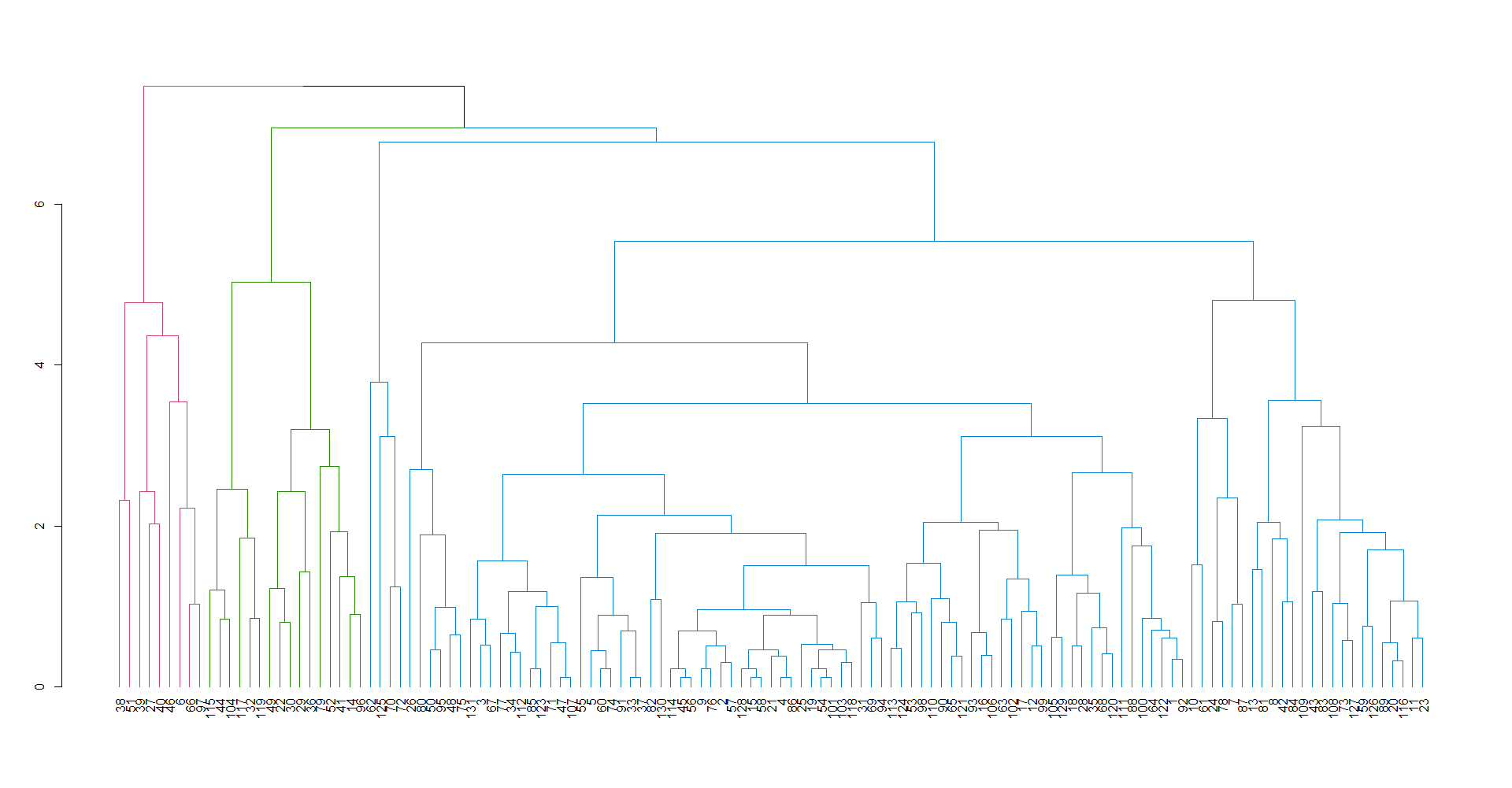
**

**Supplementary figure 3.**

**
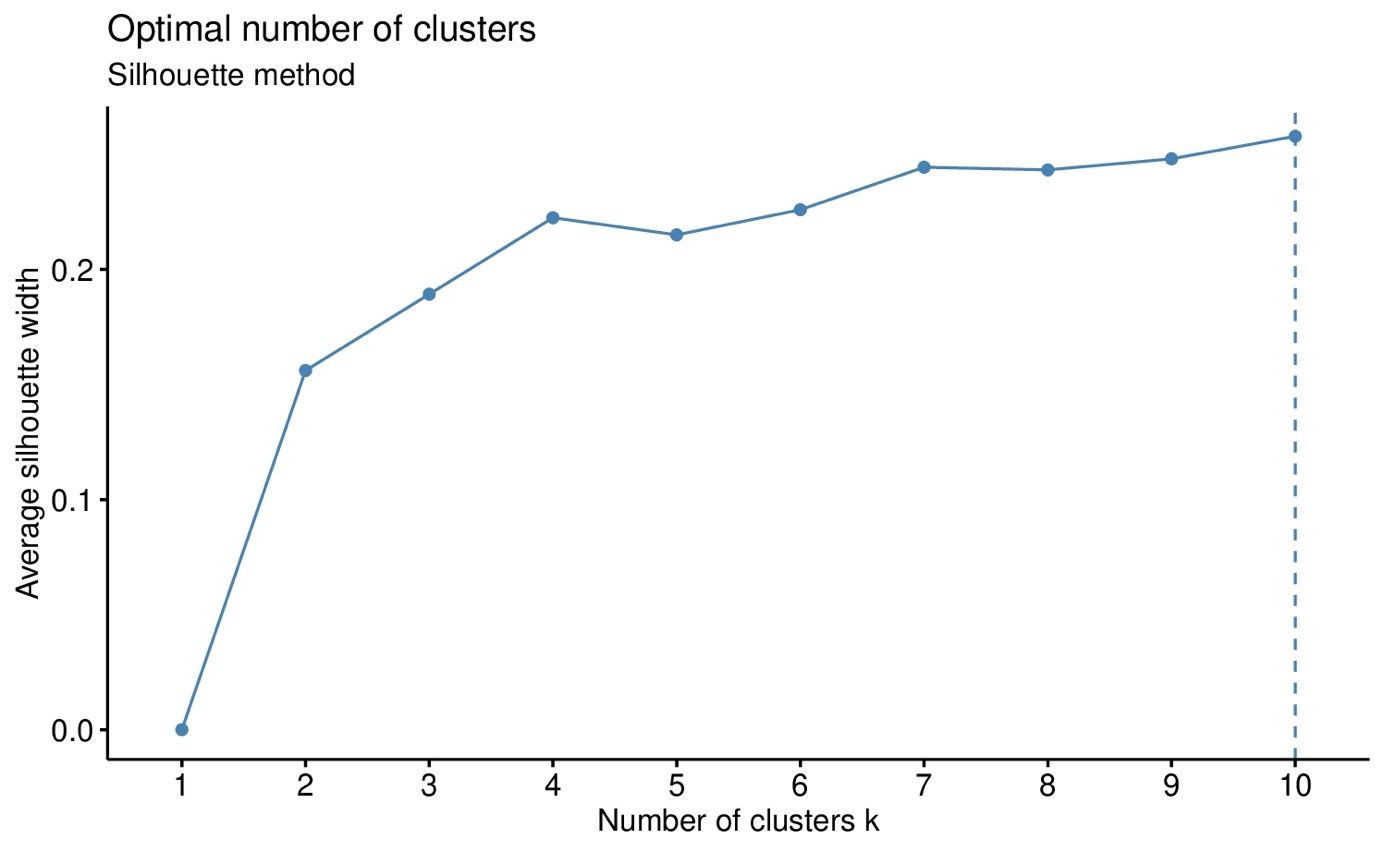
**

**Supplementary figure 4.**


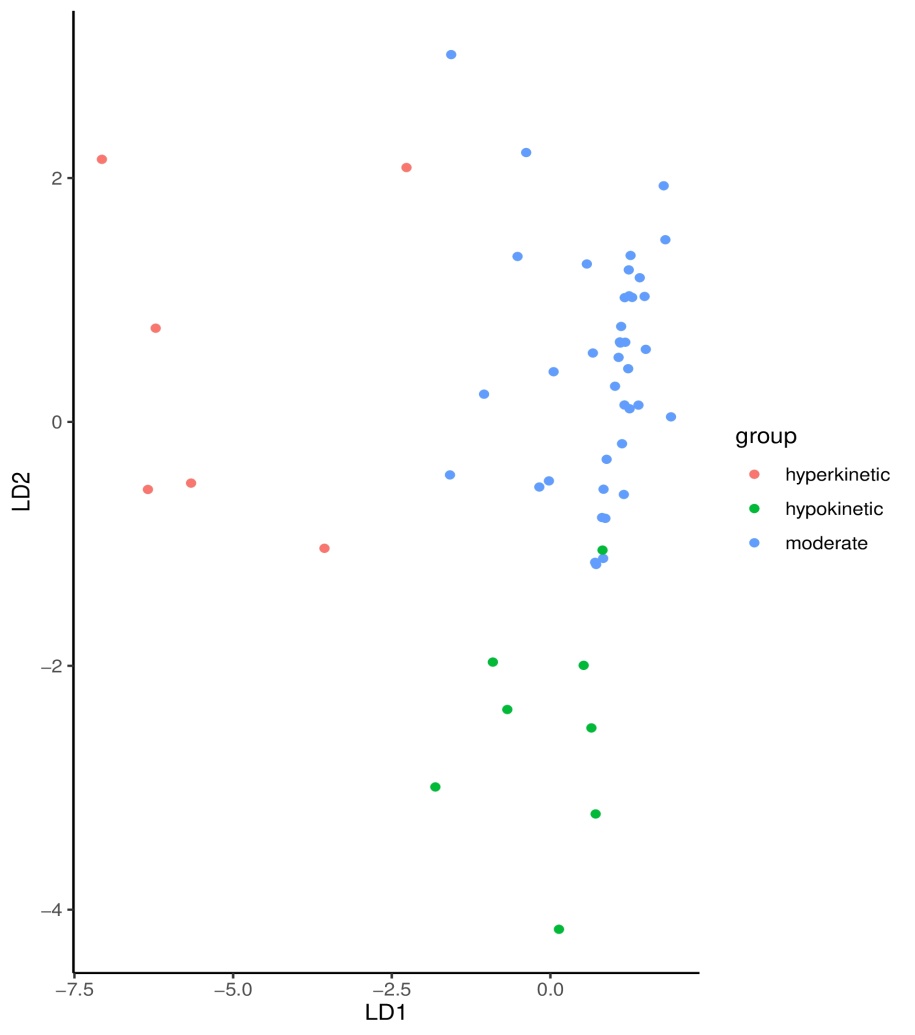


*3.2. Prevalence and overlap of sensorimotor abnormalities in schizophrenia patients (n=119)*

            The highest prevalence (115 subjects, 97%) was detected for NSS (supplementary table 3). In addition, 39 (32.8%) subjects were defined to satisfy SAS criteria for parkinsonism, while 32 subjects (26.9%) had akathisia according to BARS criteria. Also, 29 subjects (24.4%) were defined as having catatonia according to NCRS (supplementary figure 5). Finally, nine subjects (7.6%) satisfied TD criteria of Schooler-Kane, 117 patients (98.3%) satisfied criteria for at least one sensorimotor abnormality, two patients (1.7%) fulfilled criteria of all five neuromotor scores. The largest overlaps of patients fulfilling cut-off criteria for a distinct sensorimotor category were found between NSS and parkinsonism (n=39, 32.8%), NSS and akathisia (n=32, 26.9%), NSS and catatonia (n = 28, 23.5%), catatonia and parkinsonism (n=13, 10.9%) and between catatonia and akathisia (n=11, 9.2%) (supplementary table 3, figure 5 and figure 6).

**Supplementary table 3.** Number of schizophrenia patients with overlapping sensorimotor dysfunction.

| Number of patients overlapping in two sensorimotor scores | | | | |
| --- | --- | --- | --- | --- |
| AIMS & BARS | AIMS & NCRS | AIMS & NSS | AIMS & SAS | NSS & SAS |
| **5** | **4** | **8** | **3** | **39** |
| BARS & NCRS | BARS & NSS | BARS & SAS | NCRS & NSS | NCRS & SAS |
| **11** | **32** | **10** | **28** | **13** |
| Number of patients overlapping in three sensorimotor scores | | | | |
| AIMS & BARS & NCRS | AIMS & BARS & NSS | AIMS & BARS & SAS | AIMS & NCRS & NSS | AIMS & NCRS & SAS |
| **3** | **5** | **2** | **4** | **2** |
| AIMS & NSS & SAS | BARS & NCRS & NSS | BARS & NCRS & SAS | BARS & NSS & SAS |  |
| **3** | **11** | **5** | **10** |  |
| NCRS & NSS & SAS |  |  |  |  |
| **13** |  |  |  |  |
| Number of patients overlapping in four sensorimotor scores | | | | |
| AIMS & BARS & NCRS & NSS | AIMS & BARS & NCRS & SAS | BARS & NCRS & NSS & SAS | AIMS & NCRS & NSS & SAS | AIMS & BARS & NSS & SAS |
| **3** | **2** | **5** | **2** | **2** |

Data represent the number of schizophrenia patients satisfying criteria for more than one sensorimotor abnormality, *e.g. the number of patients satisfying* *AIMS and BARS criteria was n = 5*.

Abbreviations: AIMS=Abnormal involuntary movement scale, BARS=Barnes Akathisia Rating Scale, NCRS=Northoff Catatonia Rating Scale, NSS= Neurological Soft Signs Scale, SAS=Simpson-Angus-Scale.

**Supplementary figure 5.**

**
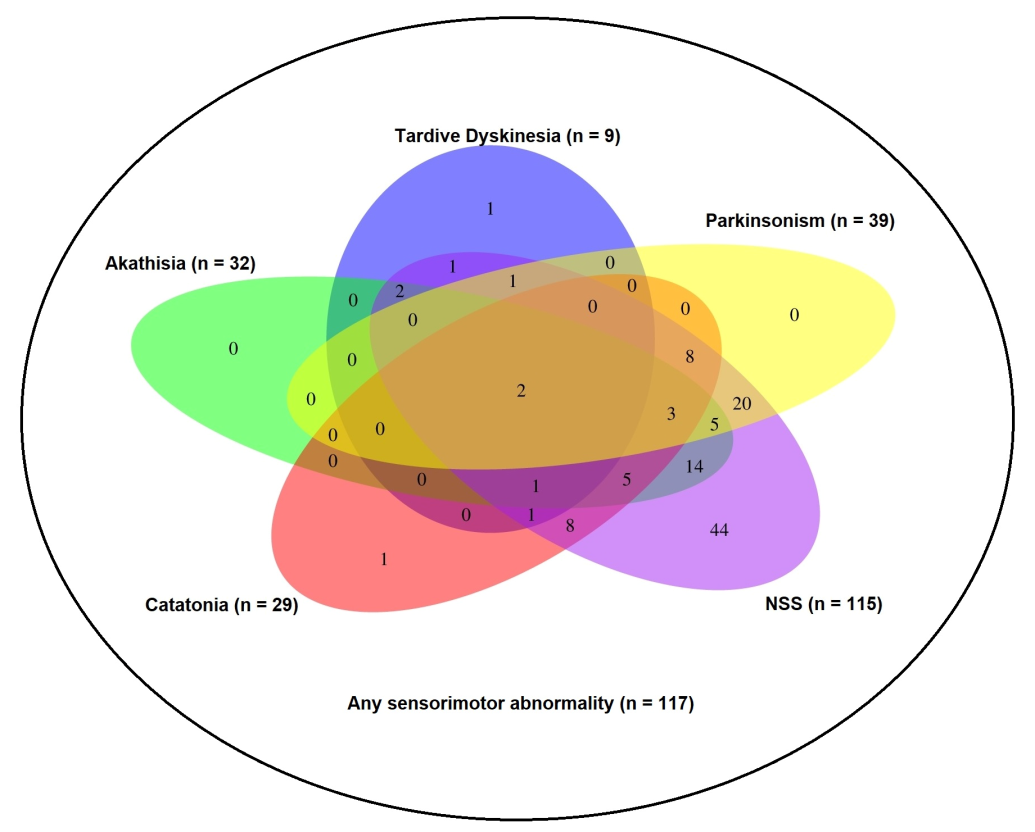
**

**Supplementary figure 6.**

**
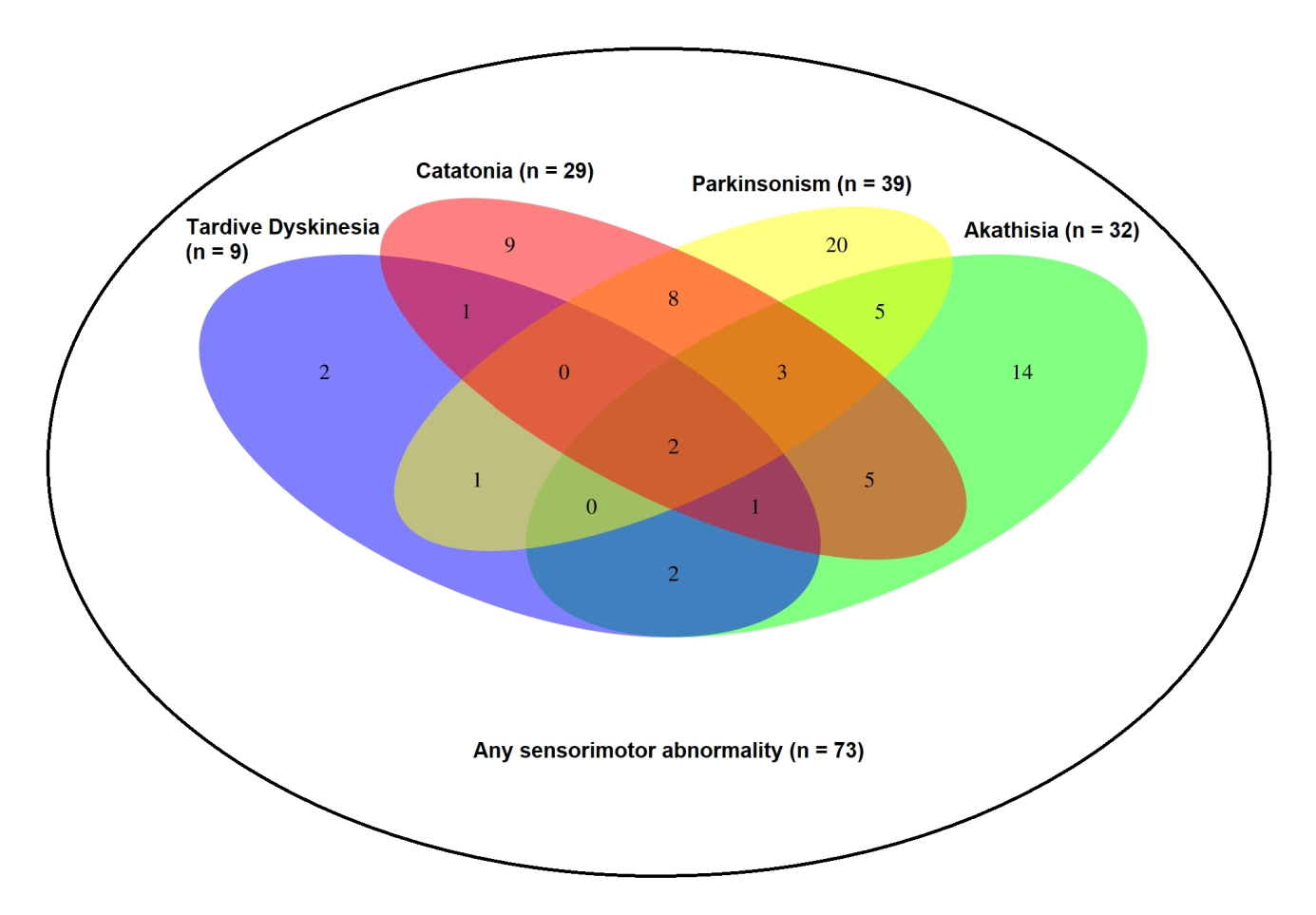
**

*3.3. Catatonia, parkinsonism, psychomotor slowing and negative symptoms in schizophrenia patients*

In the schizophrenia sample (n=119), there was no significant association between SAS and NCRS total scores (r: 0.155, p=0.099) or psychomotor slowing (BPRS item #13) and SAS (r: 0.535, p=0.06) after controlling for age, gender, OLZ and PANSS-N scores. We found a significant association between psychomotor slowing (BPRS item #13) and NCRS total scores (r: 0.738, p=0.032) after controlling for age, gender, OLZ and PANSS-N scores. We found a significant correlation between PANSS-N and SAS total (r: 0.253, p=0.006), NCRS total (r: 0.259, p=0.005) and BPRS item #13 (r: 0.581, p<0.001) scores after controlling for age, gender, and OLZ. The relationship between PANSS-N scores and SAS total, NCRS total and BPRS item #13 scores survived the Bonferroni correction for multiple testing (p=0.0125). Except for glabella sign (p=0.003), there was no significant association between OLZ and SAS or AIMS scores (according to Pearson correlation; supplementary fig. 7).

**Supplementary figure 7.**


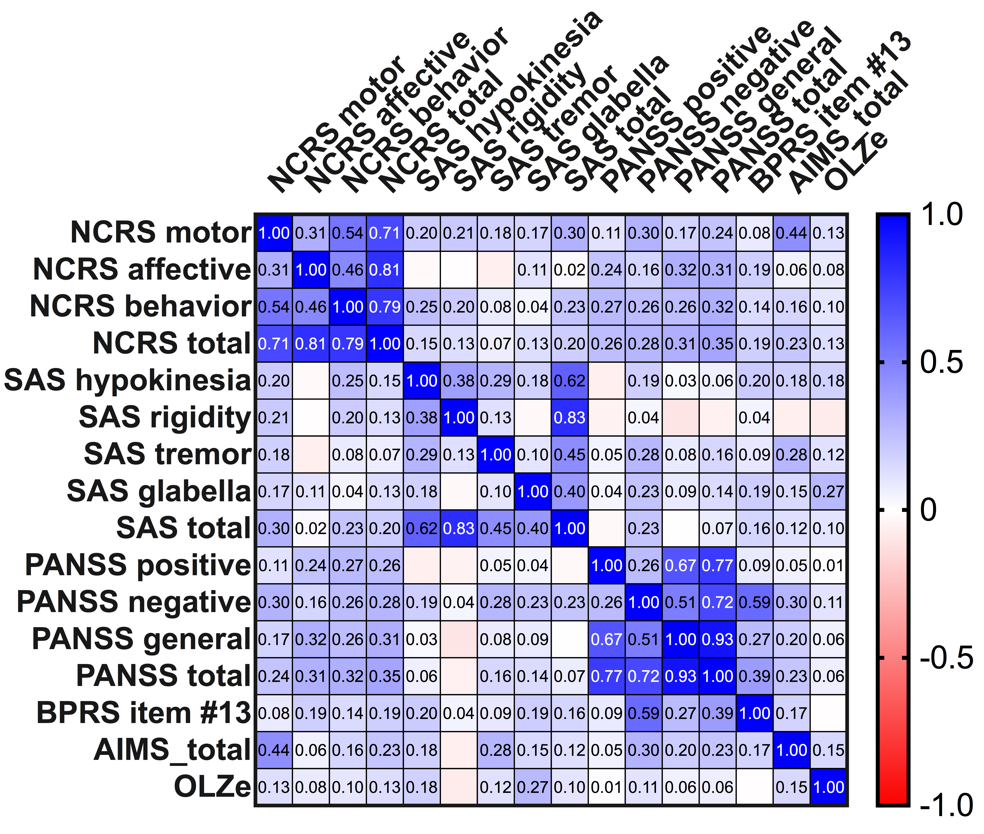


*3.4. Clustering of schizophrenia patients*

Results from the hierarchical cluster analysis showed that the 119 schizophrenia patients are optimally clustered into three subgroups (supplementary figure 8). The first cluster included 100 schizophrenia patients (84.0%), the second cluster ten schizophrenia patients (8.4%) and the third cluster nine schizophrenia patients (7.6%) (s. supplementary figure 8). The results of silhouette analysis to validate our visual choice of three clusters supported this choice (s. supplementary figure 9).

**Supplementary figure 8:**


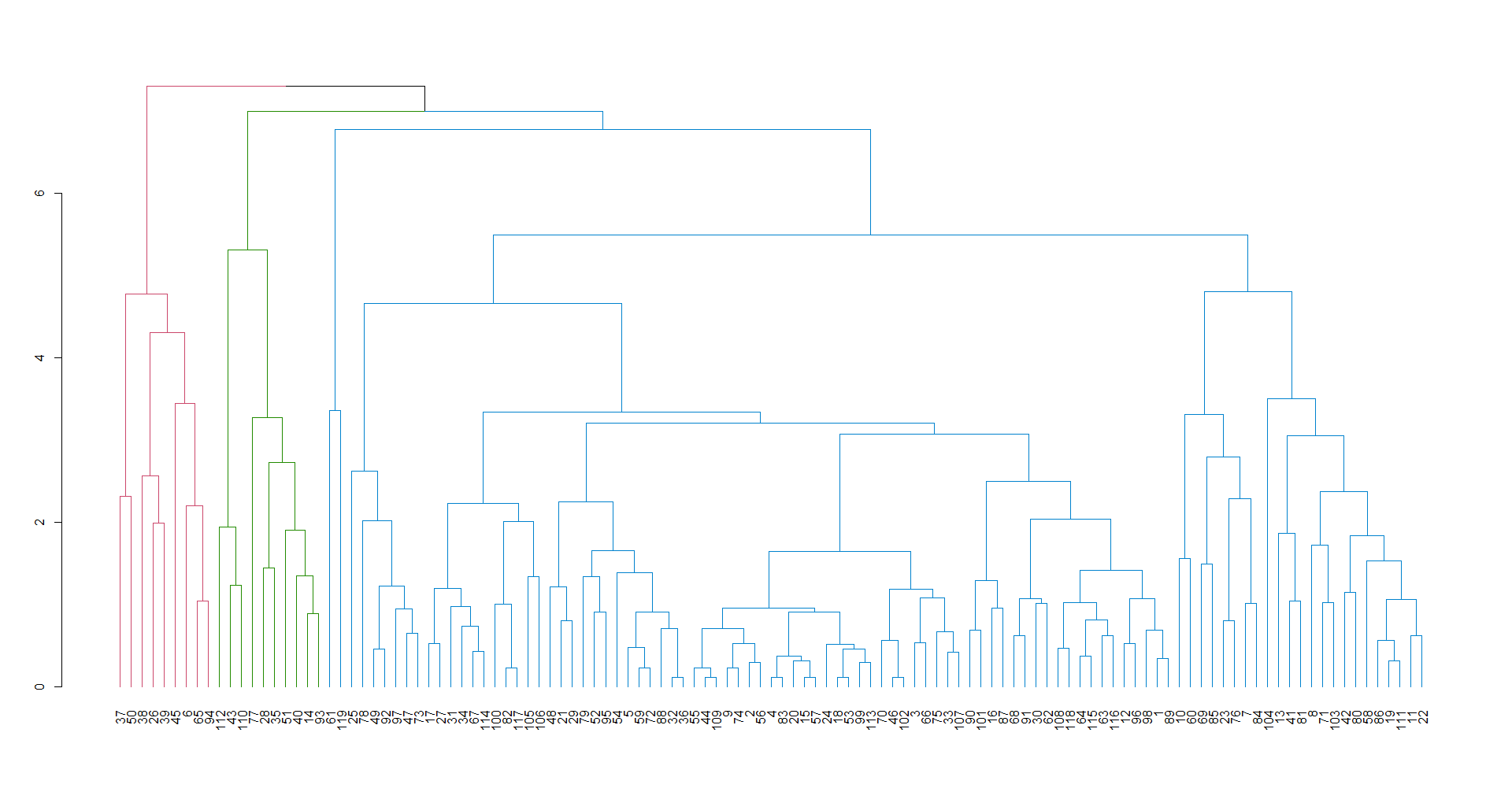


**Supplementary figure 9:**


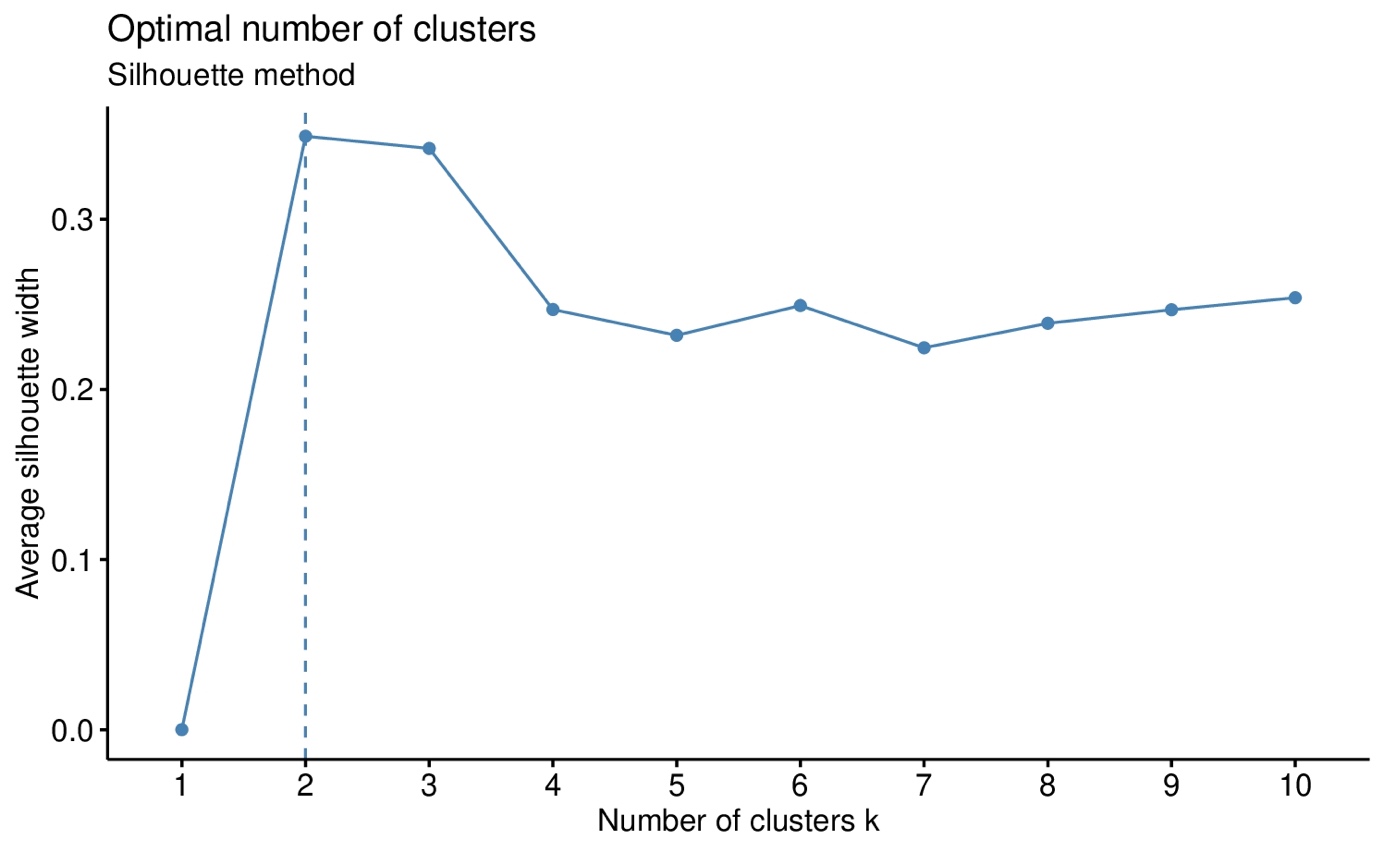


*3.5. Comparison between sensorimotor clusters on sensorimotor functioning in schizophrenia patients*

Visual comparison of standardized Z-scores showed that subjects in the first cluster exhibit below average sensorimotor abnormalities in all five sensorimotor categories, thus this cluster was labeled “moderate” (s. supplementary table 4 and supplementary figure 10). Subjects in the second cluster presented high NSS and SAS as well as below average Z-scores in the remaining sensorimotor categories, resulting in the label “hypokinetic” (s. supplementary table 4). Subjects in the third cluster showed predominant elevations in AIMS and BARS. While Z-scores in the remaining sensorimotor categories remained elevated, they were inferior to those in the hypokinetic cluster. This third cluster was labeled “hyperkinetic”. The DFA results showed two discriminant functions delineating 80.99% and 19.01% of the variance, respectively. The model accuracy in predicting subjects grouping was 0.96. The strongest negative coefficient in function 1 (LD 1) was -2.043 (AIMS), the strongest positive coefficient was 0.241 (NCRS) (s. supplementary figure 11). Results of linear DFA with a split-half of the sample to test reliability were virtually unchanged from the whole data-set (s. supplementary figure 12). Subjects grouping into the sensorimotor clusters is presented in supplementary table 4.

| **Supplementary table 4.** Comparison between the three sensorimotor clusters across distinct sensorimotor abnormalities employing Z-scores in *schizophrenia sample*. Data are given as mean. | | | |
| --- | --- | --- | --- |
|  | Cluster |  |  |
|  | Moderate (n = 100) | Hyperkinetic (n = 9) | Hypokinetic (n = 10) |
|  |  |  |  |
| AIMS | -0.26627666 | 3.04854721 | -0.08092589 |
| BARS | -0.0600884 | 0.9199663 | -0.2270857 |
| NCRS | -0.02903164 | 0.34487798 | -0.02007374 |
| NSS | -0.2566289 | 0.7165281 | 1.9214136 |
| SAS | -0.1767748 | 0.3576493 | 1.4458640 |

**Abbreviations:** AIMS = Abnormal Involuntary Movement Scale. BARS = Barnes Akathisia Rating Scale. NCRS = Northoff Catatonia Rating Scale. NSS = Neurological Soft Signs Scale. SAS = Simpson Angus Scale.

**Supplementary figure 10:**


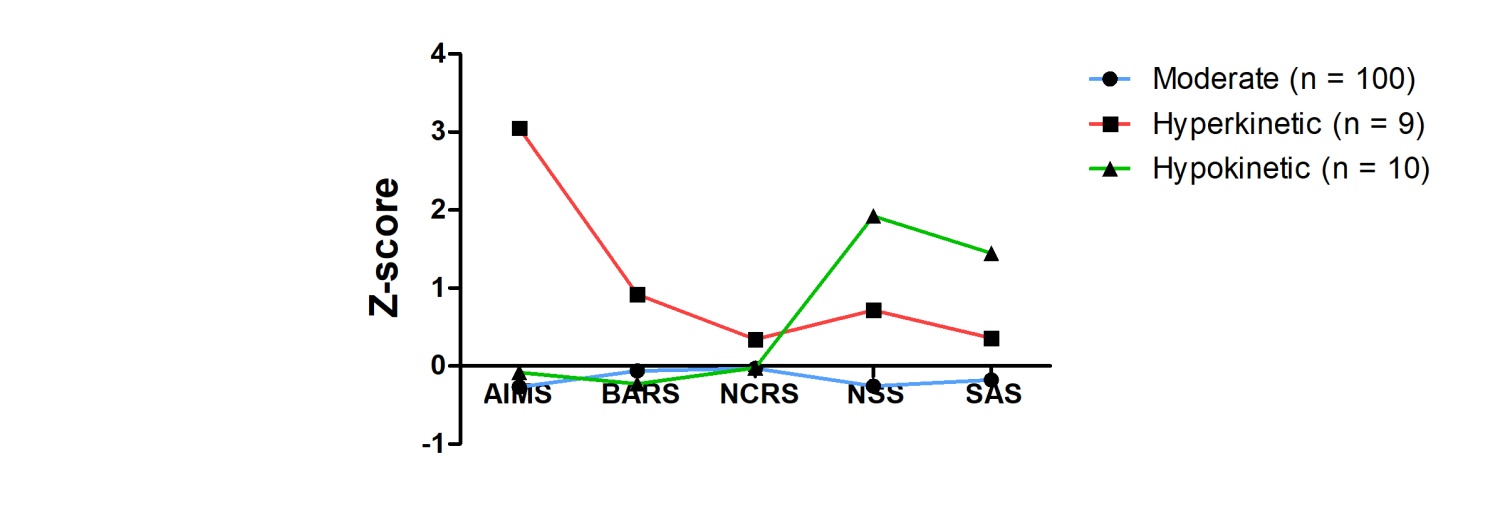


**Supplementary figure 11:**


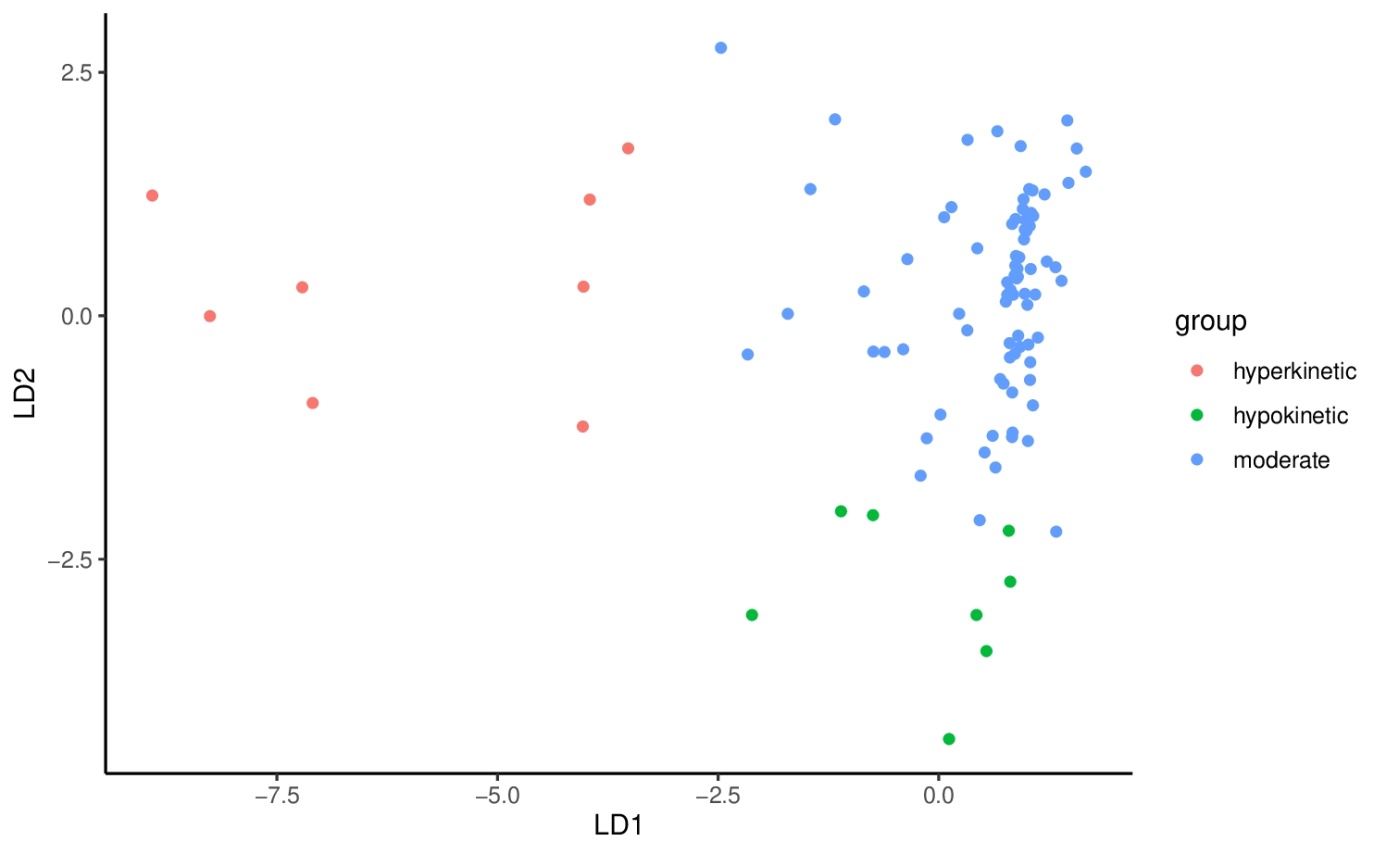


**Supplementary figure 12:**


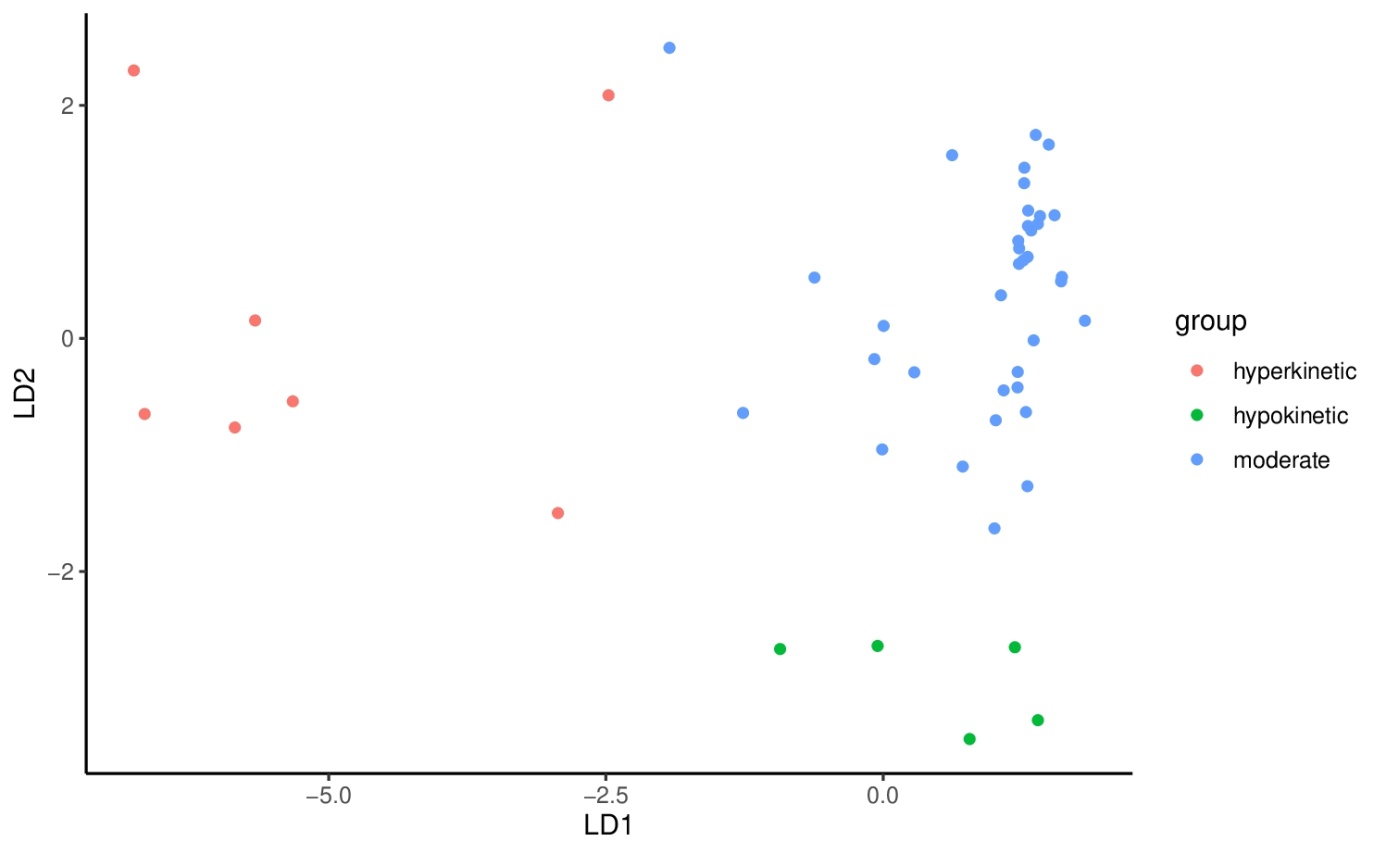


*3.6. Demographic, functional, cognitive and psychopathological correlates of the three sensorimotor clusters in schizophrenia patients*

Detailed statistics on demographic, functional, cognitive and psychopathological variables within the three sensorimotor clusters are summarized in supplementary materials. First, according to ANCOVA (after controlling for age, sex, education, medication) and post-hoc t-test, TMT appears to be increased in hyperkinetic and hypokinetic cluster, respectively, when compared to moderate cluster (s. supplementary table 5). ANCOVA for GAF, DSST and PANSS total remained non-significant after controlling for age, sex, education, medication. ANOVA revealed significant differences between the three clusters in age and DOI. Yet, ANOVA did not show any significant differences between the three clusters in sex, OLZ and education. Second, linear regression analyses showed a significant relationship between composite sensorimotor score of the moderate cluster and OLZ, GAF and PANSS total score (supplementary table 7). All three associations remained significant after adjusting α-significance level for multiple testing (p=0.05/9 = 0.005).

**Supplementary table 5.** Descriptive statistics of the three sensorimotor clusters and group-wise comparison across demographic, functional, cognitive and psychopathological variables *in schizophrenia sample (n = 119)*. Data are given as mean (SD).

|  | Cluster |  |  | | | | |
| --- | --- | --- | --- | --- | --- | --- | --- |
|  | Moderate  (n = 100) | Hyperkinetic  (n = 9) | Hypokinetic  (n = 10) | Df | F-value/ X² | p-value | Post-hoc pair-wise t-test |
| Diagnosis distribution | SZ=100 | SZ=9 | SZ=10 | - | - | - | - |
| Age* | 37.46 (10.65) | 43.11 (14.97) | 50.3 (12.15) | 2 | 6.74 | **0.002** | Hyperkinetic vs. Hypokinetic p = 0.49  *Hyperkinetic vs. Moderate* p = 0.44  *Hypokinetic vs. Moderate* ***p = 0.0021**** |
| Sex°(m/f) | 56/44 | 7/2 | 3/7 | 2 | 5.80 | 0.054 | - |
| Education* | 13.07 (2.98) | 13 (1.32) | 12.9 (3.21) | 2 | 0.017 | 0.983 | - |
| OLZe* | 17.70 (10.10) | 22.03 (12.54) | 17.37 (9.56) | 2 | 0.759 | 0.47 | - |
| DOI* | 9.03 (10.08) | 17.67 (10.94) | 24.4 (11.22) | 2 | 12.34 | **<0.001** | Hyperkinetic vs. Hypokinetic p = 0.46  *Hyperkinetic vs. Moderate* p = 0.05  *Hypokinetic vs. Moderate* ***p < 0.001**** |
| GAF^#^ | 70.39 (17.24) | 61.11 (14.53) | 64 (17.13) | 2 | 2.35 | 0.100 | - |
| DSST^#^ | 52.2 (27.67) | 43.89 (17.68) | 28.5 (9.24) | 2 | 2.16 | 0.120 | - |
| TMT-B^#^ | 101.41 (53.97) | 162.22 (70.85) | 214.1 (92.62) | 2 | 13.22 | **<0.001** | Hyperkinetic vs. Hypokinetic p = 0.176  *Hyperkinetic vs. Moderate* **p = 0.011**  *Hypokinetic vs. Moderate* ***p < 0.001**** |
| PANSS^#^ | 66.19 (21.41) | 78.00 (13.61) | 72.00 (19.04) | 2 | 1.57 | 0.212 | - |

**Abbreviations:** SZ=schizophrenia patients. SZA=schizoaffective patients. SZT=schizotypal disorder patients. DOI=Duration of Illness. GAF = Global Assessment of Functioning. DSST=Digit Symbol Substitution Test. TMT-B = Trail-Making-Test B, measured in seconds. PANSS = Positive and Negative Syndrome Scale. Df = degrees of freedom. m/f = male/female. X² = Chi-squared.

*The F- and p-values were obtained using analysis of variance (ANOVA). SD = standard deviation. Significant p-values after ANOVA or post-hoc pair-wise t-test are indicated in bold. Significant p-values that survived the Bonferroni correction (*p*=0.005) are highlighted with asterisk.

^°^ X² values were obtained using Chi-Square test.

^#^The F- and p-values were obtained using two-way analysis of covariance (ANCOVA) adjusted for age, gender, education and medication. SD = standard deviation. Significant p-values after ANCOVA or post-hoc pair-wise t-test are indicated in bold. Significant p-values that survived the Bonferroni correction (*p*=0.005) are highlighted with asterisk.

**Supplementary table 6.** Results of linear regression between composite sensorimotor (CSM) score and clinical variables in *schizophrenia sample* (n = 119).

| **Hyperkinetic cluster (n = 9)** | Adjusted R² | F-statistic | p-value |
| --- | --- | --- | --- |
| CSM score ~ PANSS total | -0.1104 | F(1,7) = 0.2049 | 0.665 |
| CSM score ~ TMT-B | -0.1335 | F(1,7) = 0.05789 | 0.817 |
| CSM score ~ DSST | -0.09781 | F(1,7) = 0.2872 | 0.609 |
| CSM score ~ GAF | 0.2967 | F(1,7) = 4.375 | 0.075 |
| CSM score ~ Age | -0.1171 | F(1,7) = 0.1613 | 0.7 |
| CSM score ~ Sex | -0.118 | F(1,7) = 0.15556 | 0.705 |
| CSM score ~ Education | -0.09937 | F(1,7) = 0.2769 | 0.615 |
| CSM score ~ OLZ | -0.08767 | F(1,7) = 0.3552 | 0.57 |
| CSM score ~ DOI | -0.01075 | F(1,7) = 0.9149 | 0.371 |
| **Hypokinetic cluster (n = 10)** |  |  |  |
| CSM score ~ PANSS total | 0.04805 | F(1,8) = 0.9149 | 0.262 |
| CSM score ~ TMT-B | 0.1095 | F(1,8) = 0.9149 | 0.185 |
| CSM score ~ SDST | -0.08543 | F(1,8) = 0.2916 | 0.604 |
| CSM score ~ GAF | 0.1863 | F(1,8) = 3.061 | 0.118 |
| CSM score ~ Age | -0.1215 | F(1,8) = 0.02493 | 0.878 |
| CSM score ~ Sex | -0.1133 | F(1,8) = 0.08378 | 0.780 |
| CSM score ~ Education | 0.004466 | F(1,8) = 1.04 | 0.338 |
| CSM score ~ OLZ | 0.1862 | F(1,8) = 3.059 | 0.1184 |
| CSM score ~ DOI | -0.05393 | F(1,8) = 0.5395 | 0.484 |
| **Moderate cluster (n = 100)** |  |  |  |
| CSM score ~ PANSS | 0.1773 | F(1,98) = 22.33 | **<0.001*** |
| CSM score ~ TMT-B | 0.002973 | F(1,98) = 1.295 | 0.2579 |
| CSM score ~ SDST | 0.01936 | F(1,98) = 2.955 | 0.088 |
| CSM score ~ GAF | 0.1662 | F(1,98) = 20.73 | **<0.001*** |
| CSM score ~ Age | -0.00779 | F(1,98) = 0.2341 | 0.630 |
| CSM score ~ Sex | 0.00022 | F(1,98) = 0.02131 | 0.8842 |
| CSM score ~ Education | -0.00696 | F(1,98) = 0.315 | 0.576 |
| CSM score ~ OLZ | 0.07571 | F(1,98) = 9.109 | **0.003*** |
| CSM score ~ DOI | -5.1e-06 | F(1,98) = 0.9995 | 0.320 |

Data represent the results of linear regression between composite motor scores (calculated as mean from z-standardized AIMS, BARS, NCRS, NSS and SAS scores) and clinical correlates. The analyses were separately conducted in each cluster. Significant p-values that survived Bonferroni correction (*p*=0.005) in each cluster are highlighted with asterisk. Abbreviations: AIMS=Abnormal involuntary movement scale, BARS=Barnes Akathisia Rating Scale, NCRS=Northoff Catatonia Rating Scale, NSS= Neurological Soft Signs Scale, SAS=Simpson-Angus-Scale. * = significant p-value**.**

**Additional analyses:**

**Supplementary table 7.** Descriptive statistics of different sensorimotor scales for each of the three sensorimotor clusters (n=131).

| **Sensorimotor scale** | **Group** | **Minimum** | **25% Percentile** | **Median** | **Mean** | **75% Percentile** | **Maximum** | **Range** | **Std. Error of Mean** | **Lower 95% CI of mean** | **Upper 95% CI of mean** | **Coefficient of variation** | **SD** |
| --- | --- | --- | --- | --- | --- | --- | --- | --- | --- | --- | --- | --- | --- |
| AIMS | Hyperkinetic | 6 | 6 | 10 | 8.67 | 10 | 12 | 6 | 0.88 | 6.63 | 10.71 | 0.31 | 2.65 |
| AIMS | Hypokinetic | 0 | 0 | 0 | 0.62 | 1.25 | 3 | 3 | 0.26 | 0.08 | 1.16 | 1.64 | 1.02 |
| AIMS | Moderate | 0 | 0 | 0 | 0.46 | 0 | 5 | 5 | 0.1 | 0.27 | 0.65 | 2.22 | 1.02 |
| BARS | Hyperkinetic | 0 | 0 | 3 | 2.11 | 3 | 4 | 4 | 0.56 | 0.81 | 3.41 | 0.80 | 1.69 |
| BARS | Hypokinetic | 0 | 0 | 0 | 0.75 | 2 | 2 | 2 | 0.23 | 0.25 | 1.24 | 1.24 | 0.93 |
| BARS | Moderate | 0 | 0 | 0 | 0.81 | 1 | 5 | 5 | 0.12 | 0.57 | 1.04 | 1.55 | 1.26 |
| NCRS | Hyperkinetic | 0 | 2 | 2 | 4.22 | 8 | 12 | 12 | 1.42 | 0.94 | 7.50 | 1.01 | 4.27 |
| NCRS | Hypokinetic | 0 | 0 | 2 | 2.31 | 4 | 6 | 6 | 0.55 | 1.14 | 3.47 | 0.94 | 2.18 |
| NCRS | Moderate | 0 | 0 | 2 | 2.96 | 5 | 16 | 16 | 0.32 | 2.32 | 3.59 | 1.12 | 3.32 |
| NSS | Hyperkinetic | 12 | 21 | 29 | 25.78 | 33 | 35 | 23 | 2.91 | 19.07 | 32.49 | 0.34 | 8.73 |
| NSS | Hypokinetic | 25 | 28 | 32.5 | 33.12 | 36.75 | 47 | 22 | 1.62 | 29.66 | 36.58 | 0.20 | 6.49 |
| NSS | Moderate | 5 | 12 | 16 | 17.17 | 21 | 37 | 32 | 0.65 | 15.89 | 18.45 | 0.39 | 6.74 |
| SAS | Hyperkinetic | 1 | 3 | 3 | 3.78 | 4 | 7 | 6 | 0.66 | 2.25 | 5.31 | 0.53 | 1.99 |
| SAS | Hypokinetic | 1 | 4 | 5 | 6.44 | 9 | 13 | 12 | 0.83 | 4.67 | 8.21 | 0.52 | 3.33 |
| SAS | Moderate | 0 | 1 | 2 | 2.38 | 3 | 9 | 9 | 0.19 | 2.00 | 2.76 | 0.84 | 2 |

Abbreviations: AIMS = Abnormal Involuntary Movement Scale. BARS = Barnes Akathisia Rating Scale. NCRS = Northoff Catatonia Rating Scale. NSS = Neurological Soft Signs. SAS = Simpson Angus Scale. SD = Standard Deviation.

**References**

1. Schroder J, Niethammer R, Geider FJ, Reitz C, Binkert M, Jauss M, Sauer H (1991) Neurological soft signs in schizophrenia. Schizophrenia research 6:25-30

2. Bachmann S, Bottmer C, Schroder J (2005) Neurological soft signs in first-episode schizophrenia: A follow-up study. The American journal of psychiatry 162:2337-2343

3. Simpson GM, Angus JW (1970) A rating scale for extrapyramidal side effects. Acta Psychiatr Scand Suppl 212:11-19

4. Hirjak D, Thomann PA, Northoff G, Kubera KM, Wolf RC (2016) Ncr-skala - deutsche version der northoff catatonia rating scale (ncrs-dv) - ein validiertes messinstrument zur erfassung katatoner symptome. Der Nervenarzt:(im Druck)

5. Hirjak D, Wolf RC, Kubera KM, Stieltjes B, Thomann PA (2016) Multiparametric mapping of neurological soft signs in healthy adults. Brain Struct Funct 221:1209-1221

6. Thomann PA, Hirjak D, Kubera KM, Stieltjes B, Wolf RC (2015) Neural network activity and neurological soft signs in healthy adults. Behav Brain Res 278:514-519

7. Cuesta MJ, Sánchez-Torres AM, de Jalón EG, Campos MS, Ibáñez B, Moreno-Izco L, Peralta V (2014) Spontaneous parkinsonism is associated with cognitive impairment in antipsychotic-naive patients with first-episode psychosis: A 6-month follow-up study. Schizophrenia bulletin 40:1164-1173

8. Peralta V, de Jalon EG, Campos MS, Cuesta MJ (2013) Phenomenological differences between spontaneous and drug-related extrapyramidal syndromes in patients with schizophrenia-spectrum disorders. Journal of clinical psychopharmacology 33:438-440

9. Hirjak D, Rashidi M, Kubera KM, Northoff G, Fritze S, Schmitgen MM, Sambataro F, Calhoun VD, Wolf RC (2020) Multimodal magnetic resonance imaging data fusion reveals distinct patterns of abnormal brain structure and function in catatonia. Schizophr Bull 46:202-210

10. Wasserthal J, Maier-Hein KH, Neher PF, Northoff G, Kubera KM, Fritze S, Harneit A, Geiger LS, Tost H, Wolf RC, Hirjak D (2020) Multiparametric mapping of white matter microstructure in catatonia. Neuropsychopharmacology 45:1750-1757

11. Janno S, Holi M, Tuisku K, Wahlbeck K (2004) Prevalence of neuroleptic-induced movement disorders in chronic schizophrenia inpatients. Am J Psychiatry 161:160-163

12. Gopal S, Xu H, Bossie C, Burón JA, Fu DJ, Savitz A, Nuamah I, Hough D (2014) Incidence of tardive dyskinesia: A comparison of long-acting injectable and oral paliperidone clinical trial databases. International journal of clinical practice 68:1514-1522

13. Schooler NR, Kane JM (1982) Research diagnoses for tardive dyskinesia. Archives of general psychiatry 39:486-487
